# Diffusion‐Based Generative Model With Scaffold‐Hopping Strategy Yields Highly Potent Bioactive Molecules

**DOI:** 10.1002/advs.75674

**Published:** 2026-05-15

**Authors:** Yuwei Yang, Xiaoqing Gong, Shukai Gu, Jing Li, Bo Liu, Yanan Tian, Qianqian Zhang, Xiaojun Yao, Huanxiang Liu

**Affiliations:** ^1^ Faculty of Applied Sciences Macao Polytechnic University Macao SAR China

**Keywords:** deep learning, diffusion model, drug discovery, molecular generation, multi‐objective optimization

## Abstract

As a critical step in drug discovery, lead optimization is a profoundly complex endeavor with a notoriously high failure rate, as it necessitates the simultaneous optimization of multiple, often conflicting parameters, including physicochemical properties, drug‐likeness, synthetic accessibility, and target binding affinity. While several generative models have been proposed for lead optimization under multi‐property constraints, they still struggle to balance multi‐objective optimization with sufficient scaffold‐level exploration. To address this challenge, we present SMarT‐Diff (Scaffold‐based Multi‐property Tuning Diffusion), a generative diffusion model that achieves this balance by reinventing scaffold hopping—enabling both property optimization and structural novelty. SMarT‐Diff achieved superior performance across diverse molecular generation and optimization metrics. Notably, across both single‐target (LRRK2, HPK1, GLP‐1R) and dual‐target (GSK3β/JNK3) molecular optimization tasks, the model consistently generated drug‐like molecules exhibiting enhanced structural diversity, preserved pharmacophoric features, and high synthetic accessibility. Furthermore, wet‐lab validation of our model‐generated compounds against LRRK2 identified a highly promising candidate with an IC_50_ of 1.544 nM, which surpasses even the positive control LRRK2‐IN‐1. This result not only confirms the compound's exceptional potency but also demonstrates the strong real‐world potential of our model to drive the design and optimization of novel, highly effective drug candidates.

## Graphical abstract

SMarT‐Diff introduces a multi‐objective generative paradigm that integrates scaffold hopping with structure‐aware scoring to enable controlled exploration beyond the training distribution. The framework consistently balances drug‐likeness, synthesizes accessibility and bioactivity, yielding chemically diverse candidates with enhanced properties. Its practical utility is further supported by wet experimental validation, which demonstrated reliable identification of novel, functionally active molecules.

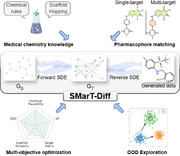

## 1 Introduction

Drug discovery has long been recognized as a time‐consuming, costly, and high‐risk process [1]. Modern artificial intelligence‐assisted drug design (AIDD) approaches have the potential to accelerate this process by empowering chemists at various stages of drug development, from lead discovery to lead optimization to drug development [2, 3, 4]. Lead optimization is a critical stage in the drug discovery pipeline, during which initial hit compounds are systematically modified and refined to develop promising drug candidates with enhanced potency, improved drug‐like properties, and favorable pharmacokinetic profiles [5, 6]. This process can be formulated as a multi‐objective optimization challenge, which entails the simultaneous maximization of drug‐likeness, synthetic accessibility, and safety profiles, alongside the enhancement of efficacy, selectivity, and key physicochemical and pharmacokinetic properties [7]. However, the conventional lead optimization process remains highly resource‐intensive, requiring multiple iterative cycles of structural design and experimental validation, and is consequently intrinsically inefficient. The incremental gains achieved through these iterative cycles are frequently non‐cumulative, meaning progress in one parameter may not sustain or contribute to overall candidate quality, ultimately constraining the entire development pipeline [8].

Unlike conventional approaches, which often rely on iterative, human‐guided modifications with limited scalability, generative models provide a systematic and bias‐mitigated strategy to explore chemical space beyond heuristic constraints [9]. In this context, the lead optimization challenge is effectively framed as a multi‐objective optimization problem, making generative models particularly well‐suited to navigate the complex trade‐offs involved. Diffusion‐based methods (e.g., TAGMol [10], PMDM [11], PMODiff [12], ECloudGen [13]) formulate molecular generation as a process of atom arrangement within protein binding pockets, guided by multiple physicochemical objectives. Large language models, such as Prompt‐MolOpt [14], MolLM [15], and MultiMol [16], achieve flexible property control through in‐context learning and integration with multi‐objective optimization algorithms. Flow‐based frameworks (e.g., MolJO [17], DrugFlow [18]) facilitate controllable multi‐property optimization by leveraging probabilistic exploration in molecular space. Meanwhile, alternative strategies like PMMG [19] employ Monte Carlo Tree Search (MCTS) to identify molecules positioned on the multi‐attribute Pareto front, representing another promising direction in the pursuit of balanced molecular design.

Despite these advances, current multi‐objective molecular optimization models still face several limitations. 3D structure‐based diffusion frameworks and flow‐based models often exhibit unstable property trade‐offs and high computational costs when optimizing multiple objectives within protein‐conditioned environments. The practical application of language models is impeded by their significant shortcomings in property controllability, balanced with topologically structured optimization. Besides, the reliance of MCTS‐based approaches on discrete exploration and post‐hoc Pareto filtering incurs high computational cost and scales poorly when extended to multi‐target‐aware molecular design. Beyond these methodological constraints, a more fundamental limitation common to current multi‐objective optimization frameworks is their poor scaffold awareness. This oversight not only hinders the effective integration of prior knowledge from known active compounds but also confines structural exploration to familiar chemical spaces, ultimately limiting their capacity to drive meaningful innovation in drug discovery.

To overcome the limitations of current models in multi‐property molecular optimization, we present SMarT‐Diff (Scaffold‐based Multi‐property Tuning Diffusion), a score‐based generative model (SGM) as one paradigm of diffusion models that reinvents Scaffold hopping for multi‐property lead optimization. It is specifically designed to generate novel molecular scaffolds while preserving critical binding poses and key interactions with the target protein. SMarT‐Diff captures the core of reference molecules based on Bemis‐Murcko (BM) scaffold [20] and incorporates the quantitative estimate of drug‐likeness (QED) [21], synthetic accessibility (SA) [22], and pharmacophore matching coefficients [23] as conditional guidance. For central nervous system (CNS) related targets, we additionally optimize blood‐brain barrier (BBB) permeability to address the unique challenges of CNS drug delivery [24]. When applied to single‐target and dual‐target drug design tasks, SMarT‐Diff demonstrates outstanding performance across multiple evaluation metrics, including effectiveness, novelty, uniqueness, and proper scaffold similarity. Additionally, we assess the real‐world applicability of our framework through experimental synthesis and in vitro assays of representative LRRK2‐targeting candidates. The encouraging inhibitory activities observed suggest that our model holds considerable promise for guiding the rational design and optimization of bioactive molecules.

## 2 Results

### 2.1 Model Overview

**FIGURE 1:**
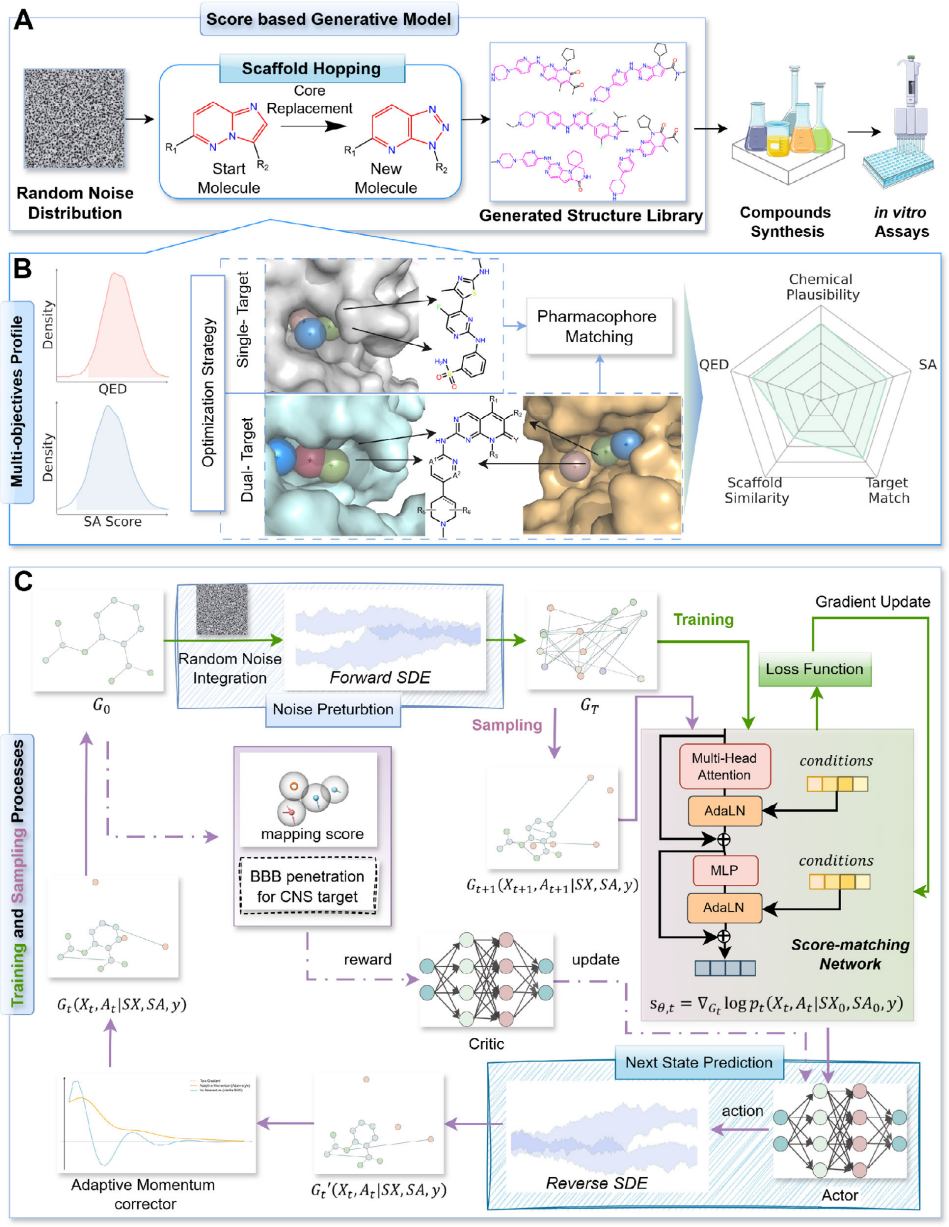
Schematic overview of the SMarT‐Diff framework for multi‐objective molecular optimization. (A) Overall workflow–from molecular generation to experimental synthesis and biological evaluation. (B) Multi‐objective criteria are considered during scaffold hopping. (C) Model architecture and pipeline, illustrating training (green arrows) and sampling (purple arrows) procedures.

Figure 1A represents the overall workflow of this work. SMarT‐Diff aims to discover novel molecules with enhanced properties by exploring beyond known chemical space. It achieves this by integrating a graph diffusion transformer (DiT) into a score‐based generative framework, which effectively captures structural patterns from training data while demonstrating strong generalization to unexplored regions [25, 26, 27]. By emulating an effective scaffold‐hopping strategy, SMarT‐Diff yields molecules with diverse and novel structures. Subsequently, to demonstrate the practical utility of our framework in real‐world drug optimization scenarios, we further validate its performance through chemical synthesis and biological activity evaluation of the generated molecules.

Trained on drug‐like small molecules, SMarT‐Diff performs five‐objective optimization by combining BM scaffold priors with property‐ and structure‐level guidance signals (QED, SA, and pharmacophore matching coefficients [23, 28]), thereby improving chemical plausibility and scaffold‐aware generation (Figure 1B). BM scaffolds derived from active molecules against specific proteins act as structural priors to guide the exploration of novel analogs within adjacent regions of the latent space (Section S1). QED and SA values are incorporated as auxiliary objectives to ensure a balanced optimization of drug‐likeness and synthetic accessibility. Meanwhile, task‐dependent optimization strategies for bioactivity are employed in SMarT‐Diff. In this study, single‐target molecular generation was evaluated on LRRK2, HPK1, and GLP‐1R, while dual‐target optimization was performed for GSK3β and JNK3 to assess the model's capacity for balanced multi‐target inhibitor design. Activity guidance is implemented by quantifying the pharmacophore similarity between generated molecules and reference single‐target and dual‐target active compounds, which improves the model's capacity to obtain candidates with high biological relevance. The pharmacophore matching coefficient between generated molecules and experimentally verified actives defines the primary reward. The detailed procedures for pharmacophore construction and matching coefficient calculation are described in Section S2.

Subsequently, details of the model architecture and pipeline were shown in Figure 1C. Across the forward stochastic differential equations (SDEs), multi‐scale noise perturbations are applied to transform the molecular structure into a noise distribution [26]. The score‐matching network is used to solve the SDE during the training process. It estimates the stochastic gradient of the joint probability distribution over all node features and edge features from whole molecules and their associated scaffolds. To enhance the learning ability of topological structures, our score‐matching network denoises unified graph tokens using Transformer layers with adaptive layer normalization (AdaLN) for structure‐aware graph transition [27]. This model architecture acquires molecular representations that simultaneously encode local connectivity patterns and internalize the coupling between structural features and property descriptors such as QED and SA scores. After minimizing the Fisher divergence between the ground‐truth and predicted scores, a robust model is ultimately obtained while avoiding adversarial optimization [29]. Once the structural distribution is learned, the generative framework employs an Advantage Actor‐Critic (A2C)‐guided sampling process as an outer optimization loop to drive pharmacophore matching optimization. The actor receives the molecular graph state and proposes structural modifications, while the critic evaluates state values and adjusts policy advantage. For CNS targets, the model also optimizes BBB permeability as an additional key property to guide generation toward CNS‐accessible candidates (Section S3). The training and evaluation performance of the BBB predictor is shown in Figure S1. At each A2C step, an inner sampling module instantiates the actor's policy signal as concrete, chemically valid edits to the molecular graph. This inner sampling module comprises a Reverse Diffusion Predictor and an Adaptive Momentum Corrector (RA), which work in concert to enable the efficient generation of out‐of‐distribution candidates [30]. Collectively, the framework efficiently explores chemically promising regions, yielding molecules that mimic scaffold hopping while achieving improved QED and SA scores.

### 2.2 Internal Evaluation of SMarT‐Diff's Generation Performance

#### 2.2.1 Ablation Studies Verify Critical Module Contributions

To validate the importance of each module, we performed ablation experiments on the LRRK2 target (Tables S2 and S3) to demonstrate how RA‐based and A2C‐guided sampling influence optimization behavior, respectively. Success rate is a key factor that reflects the proportion of molecules with a high probability of drug‐likeness and synthetic accessibility. According to Table S2, the original score‐based generator produced valid but target‐irrelevant molecules, indicating that generative capacity alone is insufficient for lead optimization. To enhance graph awareness, we replaced the denoiser with a DiT method. However, this modification caused validity to collapse to 0.419 while increasing external diversity from 17.914 to 31.792, suggesting that without structural guidance, the model drifts away from the drug‐like manifold. Thus, scaffold‐graph conditioning was introduced as the primary mechanism to enforce scaffold relevance. It restored validity to 0.953 and increased the success rate more than fivefold to 0.312. The accompanying reduction in internal diversity reflects the expected narrowing of the generative distribution following the imposition of an explicit chemotype prior. Subsequently, RA sampling acted as a chemotype‐fidelity sharpener, raising scaffold similarity from 0.430 to 0.732 without measurable loss in success rate or diversity. To further increase the success rate, edge enhancement and QED/SA incorporation played complementary but opposing roles: edge enhancement tightened scaffold similarity to a near‐collapsed 0.961, whereas the inclusion of QED/SA balanced it back to 0.794 and prevented chemotype collapse. Together, these two components pushed the success rate to 0.392 and reduced the Neighborhood Subgraph Pairwise Distance Kernel (NSPDK, which computes the Maximum Mean Discrepancy (MMD) between generated and test graphs based on node and edge features) to its minimum of 0.048. After final parameter tuning, the full model achieved the highest success rate of 0.394, while relaxing scaffold similarity to 0.654 and retaining internal diversity at 0.863. These results indicate that the four components are functionally complementary rather than merely additive.

However, the high scaffold similarity observed with the RA‐only sampling model indicates that it is still more likely to produce analogs [31]. Scaffold similarity itself is a primary design objective: values above 0.5 collapse the generator onto the reference chemotype, whereas the 0.3–0.4 window corresponds to the hopping regime conventionally associated with marketed scaffold‐hopped drugs [32, 33, 34]. To steer generation toward this regime, we introduced A2C sampling as an outer optimization loop that explicitly penalizes scaffold similarity. The ablation results in Table S3 delineate the role of each A2C component. Our framework initially leveraged an Actor‐Critic routine, which reduced the success rate from 0.394 to 0.330 but compressed scaffold similarity to 0.408, while internal diversity remained essentially unchanged (0.876 vs. 0.863). To upgrade the indicators, we e‐parameterized the actor output as a multiplicative gate, which proved catastrophic. It inflated external diversity to 23.969 but collapsed the success rate to 0.024, consistent with the destabilization of the reverse‐time SDE. The reward horizon was equally decisive: substituting cumulative discounted returns for the immediate pharmacophore reward eroded the success rate to 0.275 and uniqueness to 0.874, confirming that long‐horizon credit assignment dilutes the per‐step signal A2C is designed to amplify. Sequentially reintroducing QED and SA score progressively restored the success rate to 0.313, indicating that these objectives achieve a well‐balanced trade‐off during lead optimization. The full model, which additionally incorporates BBB permeability, attained the most favorable trade‐off, with a success rate of 0.318, the lowest external diversity in the series (18.702), and a scaffold similarity of 0.363—centered on the targeted hopping regime. These results indicate that A2C achieves a superior balance across multi‐objective optimization objectives compared to RA sampling alone, and that the improvements attributed to A2C are orthogonal to the RA sampling configuration rather than dependent upon it.

To assess whether successive components improve the model jointly across competing objectives rather than along any single axis, we benchmarked six configurations on five metrics: scaffold similarity to the reference set (targeting near 0.4), internal chemical diversity, mean QED, mean docking score, and mean SA score (Figure S2A). Radar plots of the six variants show that the final model—which optimizes four indicators—is the only configuration to expand simultaneously along all five axes, whereas single‐component variants each sacrifice one or more properties in exchange for gains on another, most notably in global scaffold exploration, local structural novelty, or predicted binding. This balanced expansion is driven specifically by the A2C loop rather than by the underlying generative framework: QED and SA distributions are essentially indistinguishable between RA and A2C sampling (Figure S2B), indicating that drug‐likeness and synthetic accessibility are determined by the core model and are largely insensitive to the choice of sampling strategy. Docking scores, in contrast, differ systematically across the three groups, with A2C sampling yielding a median score more favorable than both RA sampling and the reference actives. This demonstrates that the A2C loop selectively enhances pharmacophoric complementarity and predicted binding affinity while leaving drug‐likeness and synthetic accessibility unaffected. Together, these results establish that A2C delivers genuinely multi‐property optimization rather than trading one axis of the chemical‐space objective for another.

#### 2.2.2 Beyond Dataset Imitation: SMarT‐Diff's Adaptive Optimization Across Chemical Spaces

**FIGURE 2:**
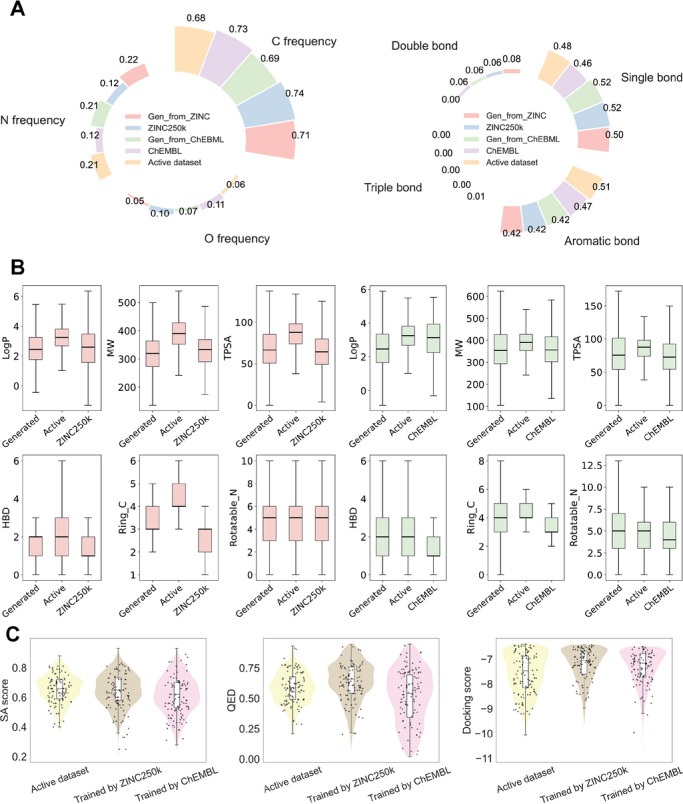
Multi‐perspective performance comparison of molecules generated by SMarT‐Diff. (A) Distributions of atom and bond types. (B) Comparison of physicochemical property distributions among generated molecules, active molecules, and training datasets (ZINC‐250k in red; ChEMBL in green). (C) Variations in key drug‐like properties across models trained on different datasets.

To examine the model's generalization ability and its sensitivity to dataset‐specific structural biases, we compared the molecules generated by SMarT‐Diff against LRRK2 when trained separately on ChEMBL [35] and ZINC‐250k [36]. The results in Figure 2 indicate that SMarT‐Diff not only assimilates the characteristic distributions of different datasets but also recalibrates its optimization trajectory to favor molecules exhibiting a more coherent balance between physicochemical realism and pharmacological relevance.

Figure 2A shows that the distribution of sampled molecular patterns closely resembles that of the bioactive reference dataset, indicating that SMarT‐Diff effectively captures dominant atom types and chemically coherent bonding motifs through scaffold hopping. The generated molecules also reproduce the intrinsic structural tendencies of their respective training datasets, reflecting a strong internalization of dataset‐specific chemical priors. Slight structural deviations, arising from dataset‐specific biases, remain within chemically reasonable bounds and reflect subtle distinctions in data composition. Collectively, the consistent recovery of meaningful substructure features across datasets highlights the model's robustness and its ability to learn transferable chemical representations.

Figure 2B shows the distributions of six physicochemical descriptors for the generated molecules. These descriptors, which reflect lipophilicity, molecular size, polarity, and flexibility, collectively capture key aspects of drug‐likeness. Their median values largely fall between those of the training and bioactive reference datasets, indicating that the generated molecules inherit dataset characteristics while gradually converging toward bioactive‐like property profiles. This intermediate property profile can be attributed, in part, to the inclusion of BBB permeability as a guiding factor, which directs the optimization toward striking a balance between hydrophobicity and polarity. Notably, generated molecules from the model trained on ChEMBL closely mirror their training data while still overlapping with the active set, demonstrating a balance between chemical fidelity and generalizable, pharmacologically relevant properties.

As shown in Figure 2C, the distributions of QED, SA score, and docking scores for the generated molecules and active compounds collectively confirm the model's multi‐objective optimization capability. Molecules generated from the ChEMBL‐trained model show broader distributions and slightly lower median QED and SA score values than those from the ZINC‐250k‐trained model. These trends resemble the intrinsic statistics of the ChEMBL database, indicating that SMarT‐Diff effectively reconstructs its underlying structural patterns. In contrast, the generated molecules show lower docking‐score distributions, suggesting that A2C sampling mediates between dataset‐learned chemical features and pharmacophore‐relevant patterns.

#### 2.2.3 Controlled Scaffold‐Level Out‐of‐Distribution Generation Preserves Binding Affinity

SMarT‐Diff is specifically designed to achieve controlled out‐of‐distribution (OOD) generation at the scaffold level, enabling the exploration of novel chemical scaffolds without sacrificing pharmacologically relevant binding affinity. Scaffold novelty was evaluated at two complementary abstraction levels following Bemis and Murcko [20]: molecular frameworks (BM scaffolds) and graph frameworks (generic scaffolds), where all atoms are reduced to carbon and all bonds to single bonds (Figure S3). Generic scaffolds offer a more stringent assessment of topological innovation by eliminating the confounding effects of bioisosteric substitutions.

Remarkably, among 10 000 molecules generated against LRRK2 (PDB ID: 8FO7), 93.96% possessed BM scaffolds, and 60.08% possessed generic scaffolds that were entirely absent from the training set (Figure S3A). Novelty rates against reference active compounds were comparable, reaching 81.87% and 60.20%, respectively, underscoring the method's strong scaffold exploration capability. Uniform Manifold Approximation and Projection (UMAP) of BM‐scaffold fingerprints (Figure S3B) revealed that generated molecules extend beyond both the training and reference active manifolds while retaining partial overlap with the latter. Furthermore, the generated scaffolds showed structural divergence characteristic of classical scaffold hopping, with nearest‐neighbor Tanimoto similarities centered at approximately 0.4 against the training set and 0.57 against reference actives (Figure S3C).

Most importantly, scaffold novelty does not come at the cost of binding affinity. Within the drug‐likeness‐filtered subset (docking score below −8.0 kcal/mol, QED > 0.6, and SA score > 0.6), molecules with a nearest‐neighbor similarity below 0.5 achieved a median Glide SP docking score of −8.57 kcal/mol (Figure S3D). This value is comparable to that of highly active reference compounds, demonstrating that favorable binding affinity is robustly preserved even as scaffold novelty increases. Collectively, these results establish that SMarT‐Diff successfully performs controlled OOD generation at the scaffold level, accessing novel chemical regions beyond the training distribution without compromising—and indeed while retaining—high binding affinity.

### 2.3 Performance of SMarT‐Diff Against Recent Scaffold Hopping‐Based Baselines

Molecular generation models that leverage scaffold hopping for practical optimization remain limited. To address this gap, we introduce SMarT‐Diff, a model that uniquely integrates topological modification with scaffold hopping under a multi‐objective optimization framework, aimed at simultaneous pharmacophore matching and drug property enhancement. In this section, we compare SMarT‐Diff's performance against recent representative scaffold‐hopping‐based baselines.

The selected baselines are described in detail below. PMDM [11] adopts a dual diffusion strategy with an equivariant dynamic kernel to stabilize the generated molecular structures. Pocket‐derived constraints are incorporated during the generation process to enhance target specificity and structural compatibility. DECOMPOPT [37] is a structure‐based decomposed diffusion model that generates molecules based on the ordered arm lists (OAL). Throughout the optimization iterations, molecular structures are decomposed to propose new arm candidates, which are subsequently evaluated via redocking to refine OAL and guide structural optimization. DRlinker [38] generates SMILES representations of molecules with specific desirable properties via a typical Transformer with policy‐based reinvent learning (RL) method. Task‐specific scoring functions are defined according to the objectives, ensuring the optimized direction. Tree‐Invent [39] integrates a topological tree into an auto‐regressive structure generative process, and performs structure editing under sub‐structural constraints. This facilitates controlled modifications while preserving critical functional motifs. DiffHopp [40] is a conditional E(3)‐equivariant graph diffusion model tailored for scaffold hopping conditioned on whole protein pockets. To further enhance the biological activity of the generated molecules, TurboHopp [41] incorporates RL techniques to optimize binding affinity scores and reduce steric clashes. It employs a target‐aware equivariant consistency model tailored for scaffold‐hopping tasks.

Table 1 presents the quality evaluation of the molecules generated by all models targeting LRRK2. Notably, SMarT‐Diff achieved a perfect novelty score of 1.0, surpassing three baselines, including DECOMPOPT (0.880), Tree‐Invent (0.920), and TurboHopp (0.936). It also demonstrated high chemical validity (approximately 0.9) while maintaining considerable uniqueness (around 0.8). On uniqueness and several other key metrics, SMarT‐Diff outperformed all other baselines and matched the performance of leading methods like PMDM and DiffHopp. Scaffold similarity to reference bioactive molecules is a central metric for assessing success in scaffold‐hopping‐based molecular optimization. SMarT‐Diff uniquely demonstrates its robustness by maintaining a scaffold similarity of approximately 0.4 while simultaneously achieving a superior structural diversity above 0.6. This balance underscores its ability to simultaneously achieve strong structural relevance and broad chemical diversity in the generated molecules. While baseline models like PMDM and TurboHopp generated structurally diverse molecules (71.8% and 61.2% Diversity), they failed to preserve scaffold similarity (nearly 0.0), indicating an inability to perform effective scaffold hopping. In contrast, SMarT‐Diff successfully maintained both diversity and similarity, demonstrating a superior capacity to simulate scaffold‐hopping strategies and capture lead optimization‐relevant experience. The drug‐related properties of the compounds generated by these models are further assessed and compared. Among all methods, SMarT‐Diff achieved the highest mean QED value of 0.640, coupled with a near‐optimal mean SA score of 0.653. This result underscores its superior capability to generate compounds that are both highly drug‐like and readily synthesizable. In contrast, while DRLinker attained the best SA score of 0.711, its average QED was considerably lower (0.059). To comprehensively evaluate model performance, we further calculated the success rate, which was defined as the proportion of generated molecules simultaneously meeting favorable QED and SA criteria. SMarT‐Diff achieved the highest success rate (0.629), substantially surpassing the best baseline model, PMDM (0.240).

**TABLE 1:** The comparison of performance between our SMarT‐Diff and scaffold‐hopping‐based baselines.

Model	Training Dataset	Validity (↑)	Uniqueness (↑)	Novelty (↑)	Scaf_sim a	Diversity (↑)	QED (↑)	SA score (↑)	Success rate b (↑)	Vina score c (↓)	BBB penetrant d (↑)
PMDM	Crossdocked2020	0.813	0.997	—	0.001	0.718	0.395	0.587	0.240	−5.815	79.08%
DECOMPOPT	Crossdocked2020	0.800	0.460	0.880	0.350	0.459	0.281	0.508	0.000	−8.034	87.50%
DRLinker	ChEMBL	0.995	0.558	1.000	0.000	0.258	0.059	0.711	0.000	−6.993	98.78%
Tree‐Invent	GuacaMol dataset	0.094	0.933	0.920	0.000	0.295	0.258	0.674	0.067	−6.561	70.12%
Turbo‐Hopp (RL)	PDBBind	0.993	—	0.936	0.000	0.612	0.583	0.631	—	−7.865	—
DiffHopp	PDBBind	0.950	1.000	1.000	0.000	0.492	0.383	0.461	0.006	−5.449	87.58%
SMarT‐Diff	ZINC250k	0.944	0.851	1.000	0.362	0.749	0.640	0.653	0.629	−7.680	96.00%

^a^ Scaf_sim quantifies scaffold similarity to the reference set, within the 0.3‐0.4 interval (optimally near 0.4), serving as a metric for effective scaffold transitions;

^b^ The success rate is defined as the proportion of generated molecules simultaneously satisfying the dual criteria of QED > 0.4 and SA > 0.6;

^c^ Vina score refers to the average docking score for the generated molecules against LRRK2 (PDB: 8FO7), calculated by AutoDock vina 1.2.0;

^d^ BBB penetrant quantifies the proportion of molecules with predicted BBB penetration scores more than 0.5.

In terms of predicted binding affinity, our pharmacophore‐guided model attained an average Vina score of −7.680, outperforming PMDM (−5.815), DRLinker (−6.993), Tree‐Invent (−6.561), and DiffHopp (−5.449), though it slightly trailed DECOMPOPT (−8.034) and TurboHopp (−7.865). The vina scores were calculated with AutoDock Vina 1.2.0 [42]. We also observed a slight trade‐off between molecular uniqueness and scaffold similarity, resulting from the stricter pharmacophore constraints of the A2C strategy, which led to a narrowing of the chemical space and increased structural resemblance to reference molecules. Furthermore, SMarT‐Diff achieved the second‐highest BBB penetration rate (96%), second only to DRLinker (98.78%). This suggests that incorporating BBB penetrability as a constraint in A2C sampling effectively guides the model toward favorable pharmacokinetic profiles without compromising pharmacophore matching. Collectively, these findings demonstrate that our model excels over baseline methods in generating structurally novel, drug‐like candidates with promising biological activity and high synthetic feasibility, underscoring the efficacy of our multi‐objective optimization framework.

### 2.4 SMarT‐Diff for Target‐Aware Multi‐Objective Molecular Optimization

#### 2.4.1 Molecular Optimization Against a Single Target

**FIGURE 3:**
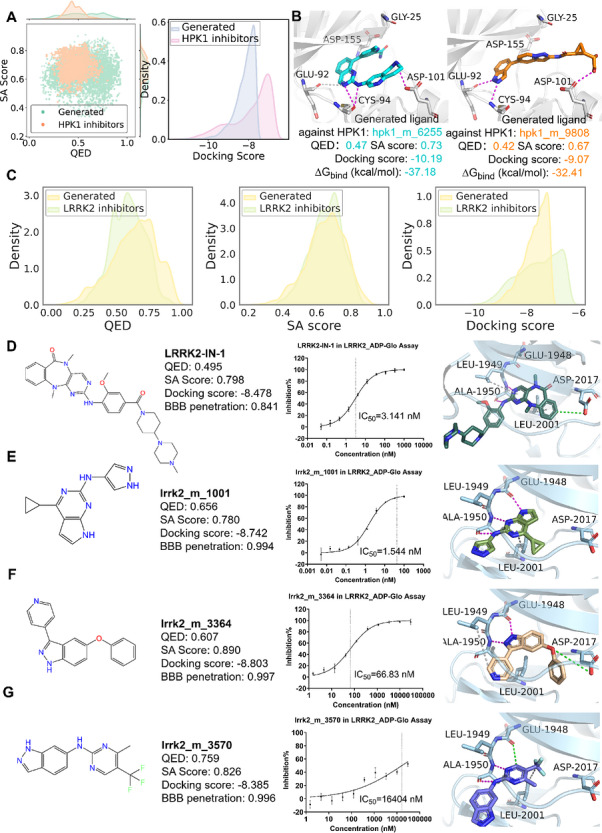
SMarT‐Diff for the design and validation of HPK1 and LRRK2 inhibitors. (A) Left: Distributions of QED and SA scores for molecules generated by SMarT‐Diff (green) and reference compounds (orange). Right: The distribution of the docking scores for the HPK1 inhibitors (purple) and generated molecules (blue) by SMarT‐Diff. (B) The interaction profiles of two representative HPK1 inhibitors generated by SMarT‐Diff (hpk1_m_6255 and hpk1_m_9808). (C) Distribution of QED, SA score, and docking scores of SMarT‐Diff‐generated (yellow) and reference (green) molecules for LRRK2. (D‐G) Chemical structures, experimentally determined IC_50_ values (ADP‐Glo Kinase Assay), and binding interaction profiles of the control inhibitor LRRK2‐IN‐1 (D, teal) and SMarT‐Diff‐generated molecules lrrk2_m_1001 (E, forest), lrrk2_m_3364 (F, smudge), and lrrk2_m_3570 (G, blue) in complex with LRRK2.

To evaluate the real molecular optimization ability of SMarT‐Diff against single‐target, three targets, including two kinases (HPK1 and LRRK2) and one GPCR (GLP‐1R), are selected. As for HPK1, the distributions of QED and SA scores of generated molecules (Figure 3A, scatter plot) are centered near 0.5 with a sparse extension below 0.3, resembling those of the reference inhibitors. This suggests that the model effectively captures a comparable trade‐off between drug‐likeness and synthetic accessibility. Additionally, distribution analysis of the docking scores (Glide SP) (Figure 3A, density plot) reveals that most generated molecules have docking scores around −8.0 kcal/mol. This represents a significant shift toward lower values compared to the reference inhibitors, suggesting an improved binding potential. Moreover, we used t‐SNE to project the optimized molecules for HPK1 and LRRK2 into a low‐dimensional space together with their bioactive reference sets, and the resulting visualization is presented in Figure S4. According to Figure S4A, the generated molecules occupy a distinct region from the reference compounds in chemical space, indicating that the model explores novel yet chemically reasonable structures while preserving drug‐like characteristics.

Based on the comprehensive multi‐attribute evaluation described in Section 4.5, two representative molecules for HPK1 are selected for further analysis. As shown in Figure 3B, compounds hpk1_m_6255 and hpk1_m_9808 exhibit similar binding modes, notably forming critical hydrogen bonds with Cys94 and water‐mediated hydrogen bonds with Asp155. These interactions are known to be essential for HPK1 activity and selectivity [43, 44]. Additionally, the pyrazole ring of hpk1_m_9808 forms an additional hydrogen bond with Glu92, which is consistent with known inhibitors. These interactions demonstrate SMarT‐Diff's ability to design molecules bearing target‐specific pharmacophoric features. The calculated binding free energies of the two generated molecules are significantly lower than those of the reference inhibitors, indicating a high binding potential with the target.

Figure 3C shows the distributions of QED, SA score, and docking scores for generated molecules against LRRK2. The majority of generated compounds cluster around a QED and a SA score of approximately 0.7, slightly outperforming the reference molecules, while docking scores concentrate around −7.5 kcal/mol, indicating stronger binding potential compared with references. Similar to HPK1, as shown in Figure S4B, the generated molecules for LRRK2 also present a different distribution in chemical space from the references. Following the selection and clustering pipeline described in Section 4.5, 10 representative generated molecules were selected (Figure S5). To further validate the practical utility of our model in real drug discovery, we selected three representative compounds (lrrk2_m_1001, lrrk2_m_3364, lrrk2_m_3570) for chemical synthesis and subsequent biological activity evaluation. Custom synthesis of previously unreported molecules involves multi‐step synthesis, purification, and rigorous characterization, which is both time‐consuming and resource‐intensive. Therefore, only three representative compounds were selected for experimental validation, a practice commonly adopted in the molecular generation field. They were synthesized by a commercial laboratory, with their structures confirmed by ^1^H‐NMR and LC‐MS analyses (Figures S6–S8). The detailed synthesized procedure can be found in Section S5. The inhibitory activity of all three compounds against LRRK2 and its G2019S mutant was confirmed in vitro using the ADP‐Glo Kinase Assay, which quantifies kinase activity by detecting ADP production (Section S6).

Figure 3D–G displays the molecular structures, experimentally determined IC_50_ values (ADP‐Glo Kinase Assay), and binding interaction profiles of LRRK2‐IN‐1 (positive control) and three synthesized molecules, lrrk2_m_1001, lrrk2_m_3364, and lrrk2_m_3570. The complete corresponding dose‐response curves are provided in Figure S9, and the quantified IC_50_ values are summarized in Table S4. The middle panels of Figure 3D–G display the inhibitory activities of the compounds against LRRK2, with IC_50_ values of 1.544 nM (lrrk2_m_1001), 66.83 nM (lrrk2_m_3364), 16.404 µM (lrrk2_m_3570), and 3.141 nM for the positive control inhibitor LRRK2‐IN‐1. Notably, lrrk2_m_1001 exhibited even superior potency over the positive control, validating our model's powerful optimization capability. Typically, three synthesized molecules adopt similar conformations to the positive control and are stably bound within the ATP‐binding pocket. The pyrazole rings in their core scaffolds form hydrogen bonds with the hinge residue Ala1950, a key amino acid critical for the activity of LRRK2 inhibitors [45]. Additionally, lrrk2_m_1001 and lrrk2_m_3364 form hydrogen bonds with the hinge residue Leu1948, whereas lrrk2_m_3570 engages in an electrophilic interaction with the same residue. Taken together with their IC_50_ values, these binding features may underline the stronger inhibitory activity observed for lrrk2_m_1001 and lrrk2_m_3364. In particular, Asp2017 forms electrostatic and hydrogen‐bond interactions with the ligands, while surrounding hydrophobic residues, such as Leu2001 and Leu1949, contribute van der Waals contacts that further stabilize binding [46]. These interactions suggest that compounds generated via SMarT‐Diff have target‐specific pharmacokinetic characteristics.

To assess cross‐target generalization, we further benchmarked SMarT‐Diff on GLP‐1R, a class B GPCR whose orthosteric pocket is markedly larger and more solvent‐exposed than the kinase targets used in the main benchmark. Without any retraining, the model generated molecules that showed improved drug‐likeness (QED) over the reference actives (median 0.575 against 0.430), while maintaining comparable synthetic accessibility (0.61 vs. 0.69; Figure S10A). The overall docking score distribution of the generated set was tighter than that of the reference actives, albeit centered at a slightly less favorable median (−7.83 vs. −8.23), reflecting the inherent challenge of sampling de novo from an underexplored chemotype space. Notably, the six top‐ranked molecules (Figure S10B) achieved docking scores ranging from −9.46 to −11.45, with three compounds scoring below −10.0. These molecules retained high drug‐likeness (QED > 0.74) and exhibited chemotypes corresponding to first‐degree scaffold hops relative to the published GLP‐1R scaffold space. Collectively, these results demonstrate that SMarT‐Diff not only performs robustly on kinase targets but also generalizes effectively to structurally and pharmacologically distinct target classes, supporting its broader applicability in lead optimization. Collectively, these findings substantiate the pharmacological potential of the generated molecules and underscore the efficacy of our generative framework.

#### 2.4.2 Dual‐Target Inhibitor Discovery Capability of SMarT‐Diff

Dual‐target drug design has attracted increasing interest in treating complex diseases such as cancer and autoimmune disorders [47, 48, 49]. An effective strategy is the pharmacophore combination approach, which leverages known structure‐activity relationships (SARs) of both targets [50]. However, this method often entails lengthy and costly trial‐and‐error cycles, primarily due to the distinct geometries and physicochemical properties of the respective binding pockets, which introduce considerable design challenges [51].

SMarT‐Diff addresses these challenges through a scaffold refinement strategy initiated by the maximum common substructure (MCS) of known inhibitors. This approach guides molecular sampling via pharmacophore matching to ensure balanced dual‐target potency, and it enhances drug‐likeness and synthetic accessibility through multi‐objective optimization. Such design flexibility allows the model to bypass the rigid pharmacophore‐fusion paradigm and instead adaptively assemble shared pharmacophoric elements into chemically feasible scaffolds. Here, we evaluated the capability of SMarT‐Diff to generate active molecules against dual‐target GSK3β and JNK3. Dual inhibitors of GSK3β and JNK3 have therapeutic potential for neurodegenerative and neuroinflammatory disorders, including Alzheimer's disease, Parkinson's disease, and ischemic stroke, but their development is often limited by poor pharmacokinetic profiles and suboptimal selectivity [52, 53, 54].

As summarized in Table S1, two inhibitor datasets were used for MCS mining. The detailed scaffold refinement process is as follows. First, active BM scaffolds are extracted from known inhibitors. Then, MCS mining of compound pairs identifies the fragments shared between inhibitors of both targets. Finally, two thousand molecules that provide the broadest coverage of these shared cores are selected as reference scaffolds to steer the generative process.

**FIGURE 4:**
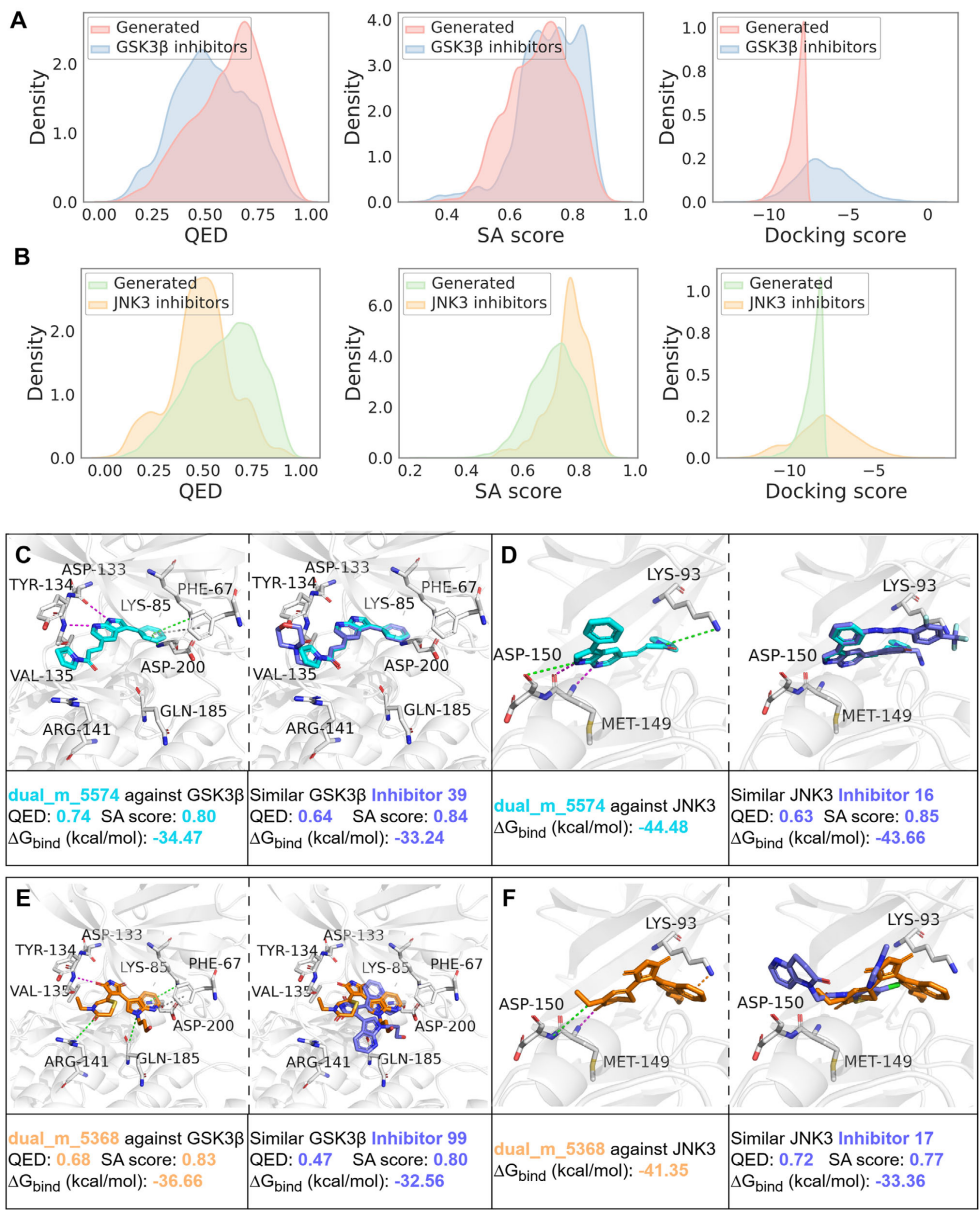
SMarT‐Diff for Dual‐Target Inhibitor Discovery against GSK3β and JNK3. (A, B) KDE plots of QED, SA, and docking scores for the generated molecules versus reference inhibitors for (A) GSK3β and (B) JNK3. (C‐F) Binding modes and MM/GBSA binding free energies for representative generated compounds dual_m_5574 (cyan; C, D) and dual_m_5368 (orange; E, F) with both targets, shown in comparison with their most similar known inhibitors (blue).

Evaluation of the generated molecules' key properties is presented in Figure 4. In panels A and B, most compounds exhibit QED scores of 0.25–0.9 and SA scores of 0.5–0.9, clustering closely with reference inhibitors and demonstrating comparable drug‐likeness and synthetic accessibility. The Kernel Density Estimation (KDE) analysis of the docking scores reveals that most generated molecules achieve dual‐target scores below −7.5 kcal/mol, with a prominent peak near −8.5 kcal/mol. This distribution indicates the model's proficiency in generating compounds with promising simultaneous binding potential for both GSK3β and JNK3. Moreover, Figure S11 shows that the generated molecules cover not only the overlapping region of both reference chemical spaces but also extend into sparsely populated areas. This demonstrates the model's ability to explore novel structural regions relevant to dual‐target inhibition. From this set, we selected the top 20 molecules per target that also ranked within the top 100 for the other. MM/GBSA calculations (Figure S12) reveal that several generated compounds achieve lower ΔG_bind_ (more favorable binding free energies) than the co‐crystallized ligands. This suggests that the generated candidates can form stable and energetically favorable interactions within both binding pockets.

Figure 4C–F present a detailed analysis of the interactions between two representative generated molecules, dual_m_5574 and dual_m_5368, with GSK3β and JNK3. Both compounds adopt binding poses highly consistent with the most similar reference inhibitors in the corresponding PDB complexes. They form multiple hydrogen‐bonding, electrostatic, and hydrophobic interactions with key residues conserved across both kinases, closely mirroring the pharmacophoric patterns of known actives. This dual‐consistent interaction pattern underscores the model's capacity to design ligands that stably engage two distinct yet complementary binding environments. The corresponding MM/GBSA binding free energies further confirm stable binding and favorable energetics for dual‐target inhibition. Overall, the potential dual‐target inhibitors identified by SMarT‐Diff exhibited key reported interactions with conserved residues across both GSK3β and JNK3 binding sites, mimicking the interaction patterns of known active ligands and supporting their promise as multi‐target kinase inhibitors.

## 3 Discussion

Lead optimization presents a formidable challenge in drug discovery, as it requires the simultaneous and balanced optimization of multiple, often conflicting parameters, such as physicochemical properties, drug‐likeness, synthetic accessibility, and target binding affinity. In this study, we proposed SMarT‐Diff, a scaffold‐constrained generative framework tailored for multi‐objective molecular optimization. The results demonstrate that SMarT‐Diff not only preserves critical pharmacophore features but also explores novel scaffolds with desired drug‐like properties. This capability is further supported by the consistent binding modes observed between the generated molecules and known inhibitors. Such consistency demonstrates SMarT‐Diff's ability to generalize key pharmacophoric and spatial constraints across diverse scaffolds, thereby validating its rational design principles.

The topological structure of a molecule, defined by its atom connectivity and functional group arrangements, often plays a more fundamental role in drug design than 3D conformation. To capture this critical information, SMarT‐Diff employs a structure‐aware scoring network that integrates multi‐head cross‐attention with adaptive layer normalization within a transformer backbone. To further address the sampling inefficiency associated with complex and highly conditioned inputs, we incorporate an adaptive momentum corrector that accelerates the denoising trajectory and improves convergence stability. This strategy facilitates stable optimization by leveraging the model's parallel processing capability to simultaneously learn from BM scaffold subgraphs, synthetic accessibility, and drug‐likeness labels. Therefore, our model enhances the chemical plausibility and rationality of optimized compounds. After incorporating pharmacophore‐matching‐guided A2C sampling, SMarT‐Diff enabled the designed inhibitors to transcend existing compound libraries while retaining potent inhibitory activity. These results highlight the ability of SMarT‐Diff to explore broader chemical space while simultaneously improving activity and enhancing structural novelty.

Across diverse evaluation settings, SMarT‐Diff demonstrated strong generalization and adaptability in multi‐objective molecular design. Comparative analyses using ZINC250k and ChEMBL datasets show that the model effectively internalizes dataset‐specific chemical priors while overcoming data dependency to explore broader regions of chemical space. Moreover, scaffold‐level out‐of‐distribution analyses indicate that SMarT‐Diff reaches chemical regions beyond the training distribution while preserving the binding affinity essential for productive lead optimization. Compared with other scaffold‐hopping‐based lead optimization models, it achieved higher validity, uniqueness, and novelty while maintaining balanced scaffold similarity. More importantly, the model markedly increases the proportion of generated molecules that meet both drug‐likeness and synthetic accessibility criteria. The framework also successfully produced CNS‐targeting molecules featuring favorable BBB permeability and lower predicted binding free energies. Overall, SMarT‐Diff achieved consistently superior performance across a range of generation tasks, including single‐target (LRRK2, HPK1, GLP‐1R) and dual‐target (GSK3β/JNK3) settings. Additionally, wet‐lab validation confirmed the practical utility of our approach, with compound lrrk2_m_1001 exhibiting marked potency against LRRK2 (IC50 = 1.544 nM), surpassing the positive control, LRRK2‐IN‐1. Collectively, these results establish SMarT‐Diff as a robust and generalizable platform for multi‐objective molecular optimization and structure‐based drug discovery.

Despite its promising performance, SMarT‐Diff still faces several limitations. First, the model's reliance on existing pharmacophore hypotheses from bioactive molecules may constrain scaffold novelty. Additionally, experimental validation on the three synthesized compounds represents only an initial experimental anchor due to practical constraints inherent to medicinal chemistry campaigns. While QED, SA score, and docking score provide tractable and widely adopted proxies for drug‐likeness, synthetic accessibility, and binding affinity, respectively, they are imperfect predictors of experimental outcomes. For instance, high QED does not guarantee favorable pharmacokinetics in vivo, SA scores are based on heuristic rules that may not reflect actual synthetic feasibility, and docking scores depend heavily on the quality of the protein structure and scoring function.

In practical drug discovery, these computational metrics serve as triage tools for prioritizing candidates prior to experimental validation, rather than as definitive measures of drug potential. In our framework, QED and SA scores are used to enforce chemical feasibility and drug‐like properties during generation, while docking scores provide an initial assessment of target engagement. The strong experimental activities observed for our synthesized compounds—particularly lrrk2_m_1001, which outperformed the positive control—suggest that these computational proxies, despite their limitations, were effective in guiding the model toward biologically relevant molecules in this case. Nevertheless, we emphasize that experimental validation remains the gold standard, and our computational metrics should be interpreted as prioritization filters rather than final indicators of drug efficacy. Moreover, capturing complex multi‐target pharmacophores without predefined hypotheses remains challenging. Future extensions will focus on integrating synthetic route learning and pocket‐aware conditional guidance to enhance scaffold innovation and synthetic accessibility. Overall, this study demonstrates that combining advanced generative optimization with rational drug design principles provides a viable path toward accelerating multi‐objective molecular discovery.

## 4 Experimental Methods

### 4.1 Datasets Information

Two datasets, ChEMBL and ZINC‐250k, are used to train the SMarT‐Diff. For the ChEMBL database, we filtered out the molecules with a molecular weight larger than 600 and AlogP > 5. The subset contains 1 254 000 molecules with nine atom types and a maximum of 48 heavy atoms in each molecule. 226 204 compounds were randomly selected for validation. Since ChEMBL is a manually curated database of bioactive molecules with drug‐like properties, generative models trained based on these molecules would facilitate learning the probability distribution of active molecular substructures. ZINC‐250k is a subset of the ZINC database that contains 250 000 drug‐like molecules with nine atom types, and the maximum number of heavy atoms in a molecule is 38. A total of 24 887 compounds were allocated to validate generative performance. Moreover, we construct the reference datasets of specific targets by using the active molecules from the BindingDB dataset [55]. In this work, we applied and evaluated our model against several targets: LRRK2, HPK1, GLP‐1R, and GSK3β/JNK3. The details of reference data about these targets are summarized in Section S1.

### 4.2 Molecular Representation

This section mainly introduces molecular representation and notations in this work. Each molecule is represented as a one‐hot feature matrix $X\;\in {\left\{0,1\right\}}^{N\times M}$ and an adjacency tensor $A\in {\left\{0,1\right\}}^{N\times N\times R}$. Here, *N* denotes the maximum number of atoms per molecule in the dataset. The parameter *M* denotes the number of atom types, while *R* denotes the number of bond types, including single, double, triple, and virtual bonds. To maintain consistent dimensions across all samples, virtual node padding is applied to both *X* and *A*. Virtual bonds are introduced to represent the absence of a connection between atoms, thereby addressing sparsity in the molecular graph's adjacency tensor. Additionally, BM scaffolds of molecules are extracted and included in the dataset to enable SMarT‐Diff to capture the full spectrum of scaffold‐related relationships. Each BM scaffold is similarly represented as a one‐hot feature matrix $SX\;\in {\left\{0,1\right\}}^{N\times M}$ and an adjacency tensor $SA\in {\left\{0,1\right\}}^{N\times N\times R}$.

### 4.3 Model Architecture

Inspired by the Graph Diffusion via the System of SDEs (GDSS) framework [56], we adopt a Score‐based Generative Model (SGM) to ensure stable and computationally efficient sample generation. The forward stochastic differential equation (SDE) introduces multi‐scale noise perturbations, progressively transforming structured molecular data toward a noise distribution. To better capture chemical complexity, node and edge features are modeled separately using distinct SDE formulations. The reverse process solves these SDEs through a score‐matching network that estimates the stochastic gradients of the joint distribution over node features, edge features, and their conditioning signals. In this study, we introduce a variation of Diffusion Transformer (DiT) as the core structure‐aware scoring module, providing a strong inductive bias for learning molecular scaffolds and pharmacologically relevant patterns [27, 57]. Model training minimizes the Fisher divergence between ground‐truth and predicted scores, yielding a well‐calibrated scoring network for generations. Subsequently, the model follows a predictor‐corrector sampling scheme to construct the core RA sampling trajectory. This trajectory is then embedded within an A2C optimization framework, where pharmacophore‐matching scores serve as the guiding signal, steering the sampler toward molecules with enhanced structural and functional quality. The following subsections detail these architectural components.

#### 4.3.1 Generative Framework of SmarT‐Diff

Here, we employ a score‐based diffusion model as the generative framework to sample out‐of‐distribution objects. To ensure accurate score estimation, we perturb the data at multiple noise levels, striking a balance between exploring the perturbed space and maintaining fidelity to the original data distribution. Specifically, the forward perturbation process gradually transforms a molecule *x*_0_ into a noisy sample *x_t_* as defined by the following SDE:

(1) $$\begin{array}{c} d\;x_{t}=f\left(x_{t},t\right)dt+g\left(t\right)dw_{t} \end{array}$$

which is constructed by the drift coefficient*f*(*x_t_*,*t*), the diffusion coefficient *g*(*t*) and an infinitesimal white noise *dw_t_* that is extracted from a standard Brownian motion. The goal of the generative model is to learn a time‐dependent score function *s*_θ_(*x_t_*,*t*). During sampling, the system of reverse‐time SDEs is used as a generative model by simulating the system backward in time. In this study, we use the Variance Preserving SDE (VP SDE) to add noise perturbations for node features and the Variance Exploding SDE (VE SDE) for edge features.

##### 4.3.1.1 VP‐SDE for Node Features

Following the previous approach [56], the noise perturbation of node features is modeled using a VP‐SDE:

(2) $$\begin{array}{c} d\;x_{t}=-\frac{1}{2}\beta \left(t\right)x_{t}dt+\sqrt{\beta \left(t\right)}dw_{t} \end{array}$$

Here β(*t*) controls the noise schedule and *w_t_* is standard Brownian motion.

The corresponding reverse process is governed by the following SDE:

(3) $$\begin{array}{c} d\;x_{t}=\left[-\frac{1}{2}\beta \left(t\right)x_{t}-\beta \left(t\right)s_{\theta }\left(x_{t},t\right)\right]dt+\sqrt{\beta \left(t\right)}d{\bar{w}}_{t} \end{array}$$

This formulation enables stabilized feature diffusion without sacrificing atomic‐level semantic information.

##### 4.3.1.2 VE‐SDE for Edge Features

The noise perturbation for edge representations is modeled using a VE‐SDE:

(4) $$\begin{array}{c} \;dx=\sqrt{\frac{d\left[\sigma^{2}\left(t\right)\right]}{dt}dw}\; \end{array}$$

The corresponding reverse process for edge feature denoising (Equation 5) serves to progressively remove noise.

(5) $$\begin{array}{c} d\;e_{t}=-\frac{d\left[\sigma^{2}\left(t\right)\right]}{dt}s_{\theta }\left(e_{t},t\right)dt+\sqrt{\frac{d\left[\sigma^{2}\left(t\right)\right]}{dt}}d{\bar{w}}_{t} \end{array}$$

The use of a monotonically increasing σ(*t*) broadens the variance during this process, thereby encouraging the exploration of diverse bond patterns.

#### 4.3.2 Architecture of Score Matching Network

##### 4.3.2.1 Conditional Modulation Module

To capture both the overall distribution of molecular structures and the critical relationships between the core scaffold and its substituents, the score‐matching network is conditioned on a composite vector *y* that concatenates a continuous vector $s\in R^{k}$ and categorical label r∈{0,…,C−1}. For any condition variable *c*, the final embedding fed to the network is given by

(6) $$\begin{array}{cc} & \hat{e}\;\left(c,\;m,\xi \right)=\left(1-m\right)\;f\left(c\right)+mg\left(c\right)+\xi ,\;m∼Bernouli\left(p\right),\\ & \xi ∼N\left(0,I_{d}\right) \end{array}$$

where

(7) $$\begin{array}{c} \begin{array}{c} f\;\left(c\right)=\left\{\begin{array}{c} E_{cat}\left(c\right),\;c\in \left\{0,\text{…},C-1\right\},\\ W_{2}softmax\left(W_{1}\cdot c\right),\;c\in R^{k}, \end{array}\right.\;g\;\left(c\right)=\left\{\begin{array}{c} E_{cat}\left(C\right),\\ e_{drop}, \end{array}\right.\; \end{array} \end{array}$$

In this study, scaffold features (*SX*,  *SA*) are encoded as categorical conditional inputs, while the QED and SA scores are embedded as continuous conditional inputs. The timestep t is also treated as a special condition and encoded to a D‐dimensional representation *t* by sinusoidal timestep embedding. Consequently, the conditional score network is designed to learn:

(8) $$\begin{array}{c} s_{\theta }\left(x_{t},\;y,t\right)\approx \nabla_{x_{t}}\log p_{t}\left(x_{t}|y\right) \end{array}$$

Here, *x_t_* ​ denotes the perturbed molecular features (*X*,  *A*) of a batch, which are first projected via a linear embedding layer. This conditional mechanism thereby enhances the model's sensitivity to multi‐property constraints such as drug‐likeness and synthetic accessibility, effectively orchestrating scaffold‐aware molecular reconstruction and optimization.

##### 4.3.2.2 Structure‐Aware Module

To support scaffold‐hopping strategies, the score‐matching model in SGM must exhibit strong structural awareness, enabling it to capture how gradient variations relate to underlying molecular geometry. Accordingly, we adopt a DiT‐based architecture [58] as the foundation of our score‐matching network. Given a noisy molecular graph at timestep *t*, we first embed the node‐edge tokens into the hidden space through a linear projection:

(9) $$\begin{array}{c} H=Linear\left(X_{t}^{G}\right),\;H\in R^{N\times D} \end{array}$$

where $X_{t}^{G}$ denotes the concatenated node and edge features. The Transformer layers integrate conditional embeddings via layer‐wise modulation, allowing property‐aware feature refinement during denoising. Among these, the adaptive layer normalization (AdaLN) [57, 58] controlled by the representations of the conditions *y* transforms the hidden states *H* as *AdaLN*(*H*,  *y*). For each hidden vector h∈H:

(10) $$\begin{array}{c} AdaLN\;\left(h,\;y\right)=\gamma_{\theta }\;\left(y\right)\;⊙\;\frac{h-\mu \left(h\right)}{\sigma \left(h\right)}+\beta_{\theta }\left(y\right) \end{array}$$

where μ(*h*) and σ(*h*) denote feature‐wise mean and variance, and γ_θ_(*y*), β_θ_(*y*) are two‐layer MLPs with SiLU activation [59]. Their first linear layers are zero‐initialized to stabilize conditioning during early training.

To better regulate residual pathways, a gated variant is used:

(11) $$\begin{array}{c} AdaLN_{gate}\;\left(h,y\right)=\alpha_{\theta }\;\left(y\right)⊙\;AdaLN\left(h,y\right) \end{array}$$

where α_θ_(*y*) shares the same architecture and initialization strategy.

The global self‐attention mechanism facilitates the learning of long‐range dependencies and multi‐center interactions, which are critical for capturing scaffold‐substituent relationships during molecular reconstruction.

After the final Transformer layers, an MLP head followed by AdaLN produces the denoised node and edge distributions at *t*  =  0:

(12) $$\begin{array}{c} \;{\tilde{X}}_{G}^{0}=AdaLN\left(MLP\left(H\right),y\right) \end{array}$$

The output is then split into node‐type and bond‐type logits:

(13) $$\begin{array}{c} \;{\tilde{X}}_{G}^{0}=\left[{\tilde{X}}_{V}^{0}∥{\tilde{X}}_{E}^{0}\right] \end{array}$$

where the first *F_V_* channels correspond to node probabilities, and the remaining *F_E_* channels represent bond assignments.

Once the score‐matching network is trained and the relevant reference information is collected, novel molecular structures can be generated by sampling around the corresponding distribution.

#### 4.3.3 Training Objectives of SMarT‐Diff

The SGM, as the generated framework of SMarT‐Diff, is trained by minimizing Fisher divergence between the ground‐truth scores and the model‐predicted scores across all noise levels. This objective ensures that the learned score‐matching network accurately estimates the gradients of the data distribution required for reverse‐time denoising. In this study, the training objective computes the $\ell_{2}$ loss independently for node and edge features, measuring the discrepancy between the model‐predicted noise gradients and the true gradients under the imposed conditional constraints:

(14) $$\begin{array}{cc} obj\_X & ≔\min_{\theta }E_{t}\left\{\lambda_{1}\left(t\right)E_{G_{0}}E_{G_{t}\left|G_{0},\;G_{s0}\right.}∥s_{\theta ,t}\left(G_{t}\right)\right.\\ & \;-\left.\nabla_{X_{t}}\log p_{0t}\left(G_{t}\left|G_{0},\;G_{s0}\right.\right){∥}_{2}^{2}\right\} \end{array}$$

(15) $$\begin{array}{cc} obj\_A & ≔\min_{\phi }E_{t}\left\{\lambda_{2}\left(t\right)E_{G_{0}}E_{G_{t}\left|G_{0},\;G_{s0}\right.}∥s_{\phi ,t}\left(G_{t}\right)\right.\\ & \;-\left.\nabla_{A_{t}}\log p_{0t}\left(G_{t}\left|G_{0},\;G_{s0}\right.\right){∥}_{2}^{2}\right\} \end{array}$$

where λ_1_(*t*) and λ_2_(*t*) are positive weighting functions and *t* is uniformly sampled from [0,  *T*].

#### 4.3.4 Core of Samplers: Reverse Diffusion Predictor and Adaptive Momentum Corrector

To boost the population of molecules with optimal drug‐like properties and synthesizability, the model requires enhanced representations of diverse atomic and bond interactions. However, the high complexity of chemical topologies imposes a substantial computational overhead, presenting a key challenge. We draw inspiration from the predictor‐corrector sampler and extend its original framework to better accommodate the feature distribution of molecular graphs, resulting in a more efficient and refined sampler. At each timestep *t*, the unified score:

(16) $$\begin{array}{c} s_{x},\;s_{a}=s_{\theta }\;\left(x_{t},t,y\right) \end{array}$$

are used to evaluate the drift *f_t_* and diffusion *g_t_* of the underlying reverse‐time SDE. The reverse‐time SDE is discretized as:

(17) $$dx=f\left(x,t\right)-g\left(t\right)g{\left(t\right)}^{T}\nabla_{x}logp_{t}\left(x\right)dt+g\left(t\right)d\bar{w}$$

When provides iteration i∈{0,…,N−1}, *x* can be updated as:

(18) $$\begin{array}{c} \begin{array}{c} \;x_{i}=x_{i+1}\;-f_{i+1}\left(x_{i+1}\right)+g_{i+1}g_{i+1}^{T}s_{\theta }\left(x_{i+1},i+1,y\right)+g_{i+1}z_{i+1} \end{array} \end{array}$$

where z∼N(0,I).

It establishes a coarse reverse‐diffusion trajectory. Subsequently, an adaptive momentum corrector refines the predicted samples by using score‐based gradients while introducing momentum and adaptive updates to improve stability and sampling efficiency [30]. Given the score gradients *g*, the adaptively computed momentum factor β is:

(19) $$\beta_{t+1}=\left\{\begin{array}{cc} \mathrm{Pro}j_{\left[0,1-\delta \right]}\left(\frac{1-\alpha \frac{∥g^{t}-g^{t-1}∥}{∥x^{t}-x^{t-1}∥}}{1+\alpha \frac{∥g^{t}-g^{t-1}∥}{∥x^{t}-x^{t-1}∥}}\right), & t\geq 2\\ 0, & k=0,\;1 \end{array}\right.$$

where *Proj*_[0, 1 − δ]_(·) :   =  *max*(0,  *min*(·, 1 − δ)) operation with a threshold δ.

According to the Gaussian noise ε∼N(0,I), x would be updated based on the trained score‐matching network *s*_θ_(*x^t^*,*t*, *y*) as follows:

(20) $$\begin{array}{c} \;x^{t+1}=x^{t}+\tilde{\alpha }m^{t}+\sqrt{2\alpha }\varepsilon ,\;\;m^{t}=\beta_{t}\;m^{t-1}+\left(1-\beta_{t}\right)s_{\theta }\left(x^{t},t,y\right) \end{array}$$

where α is a step size parameter, and make stepsize schedule as $\tilde{\alpha }=\alpha {\left(1+\beta \right)}^{2}$.

The predictor guides the reverse‐time evolution toward high‐density regions of the data manifold, while the momentum corrector provides fine‐grained structural adjustments with improved numerical stability. Together, these modifications enhance the fidelity and realism of the generated molecular graphs while reducing the computational cost of sampling.

#### 4.3.5 Reinforcement Learning Guided Sampling for Enhanced Bioactivity of Molecules

Enhancing the potential activity of molecules is a key aspect of molecular optimization. This study incorporates the A2C reinforcement learning framework into the sampling process. The reverse diffusion process, formulated as a Markov decision process, provides an appropriate stage for inserting the plug. A2C is a powerful reinforcement learning algorithm that combines policy gradient methods with value function approximations, improving learning efficiency through the simultaneous training of a policy network called Actor and a value network called Critic. During each update, the agent interacts with the environment to collect trajectories of states, actions, and reward. Several recent works on pharmacophore‐assisted molecular generation have demonstrated the practical value of incorporating pharmacophoric constraints into generative workflows [23, 60, 61]. Consistent with these advances, SMarT‐Diff adopts a similar strategy, integrating pharmacophore information as a key guidance signal during molecule generation. A pharmacophore reference dataset was constructed from active compounds of the target, and the deviation between the generated molecule's topological matching coefficient and the reference value was used as the reward signal for the A2C policy.

The Actor, implemented as a policy network, utilizes graph convolutional layers followed by a multilayer perceptron (MLP) to extract features and generate action policies. The Critic estimates the Q‐value of state‐action pairs, providing essential feedback to guide the Actor's policy refinement. The actions calibrate the scores from the diffusion model to fine‐tune the predictor's denoising steps. In the A2C sampling process, the Critic effectively adjusts the sampling strategy, removing the need for a corrector. After multiple iterations, this method enhances the model's ability and efficiency in producing molecules with higher potential activity.

For CNS‐related targets, the predicted BBB permeability was incorporated as an additional component of the reward during A2C‐guided sampling. The total reward was defined as

(21) $$\begin{array}{c} r=0.5\times \left(1.0-\left|s_{pred}-s_{target}\right|\right)+{\hat{r}}_{BBB} \end{array}$$

where *s_pred_* and *s_target_* denote the predicted and desired pharmacophore mapping scores, respectively, and ${\hat{r}}_{BBB}$ represents the normalized BBB permeability predicted by the auxiliary model.

This formulation encourages the agent to optimize both pharmacophore alignment and BBB penetration potential simultaneously during the sampling process.

### 4.4 Multi‐Property‐Guided Molecular Optimization via SMarT‐Diff

#### 4.4.1 Model Training Process

The model was trained by minimizing a denoising score matching objective under the conditional guidance of molecular properties and scaffold features. Conditional embeddings of QED, SA score, and scaffold topology were integrated during training through feature‐wise modulation. Optimization was performed using the AdamW optimizer (learning rate 5 × 10^−4^, cosine decay) with gradient clipping (max norm 1.0) and EMA parameter averaging (decay 0.999). Each batch contained 1024 molecular graphs sampled from the combined ChEMBL and ZINC250k datasets. To ensure training stability, time steps *t* were uniformly sampled and reweighed according to the noise variance. The model was trained for 500 epochs on a single NVIDIA A100 GPU.

#### 4.4.2 Molecular Generation Process

Once the structure‐aware score‐matching network is trained, molecules are generated through an RA sampler, which is embedded as the inner routine of an A2C loop. RA governs the reverse diffusion trajectory, while A2C supplies a goal‐oriented steering signal that biases this trajectory toward pharmacologically desirable regions. Notably, A2C never updates the pretrained score network; instead, it acts solely on the sampling path. This design preserves the learned chemical prior while imposing task‐specific objectives on top of it.

For each target, conditioning vectors are assembled from the BM scaffold features of the active compound set, along with their desired QED and SA values. These vectors are then injected into the score network via adaptive normalization layers to enforce target‐ and property‐aware denoising. Sampling is initialized from a Gaussian prior at the terminal diffusion time *T* and proceeds along the RA schedule. At each step, the score network outputs node‐ and edge‐level scores that parameterize the reverse‐time SDE. The predictor discretizes this SDE to yield a coarse denoised state, which the corrector then refines through adaptive‐momentum Langevin updates. The step sizes of these updates are modulated by a target signal‐to‐noise ratio (SNR), balancing deterministic guidance against stochastic exploration. The graph obtained at *t* = 0 is filtered for chemical validity and scored based on pharmacophore matching.

Within this loop, A2C treats each intermediate state as an environment state and the corresponding sampling adjustment as an action. The actor predicts a guidance action that rescales the update applied to the unified node‐edge features, steering generation toward target‐specific interaction patterns. Meanwhile, the critic estimates the expected long‐term reward and provides a variance‐reducing baseline. The resulting advantage then drives the actor updates. The primary reward is the pharmacophore‐matching coefficient. For CNS targets, a BBB permeability term is added with a weight of 0.5, encouraging brain‐penetrant structures without overwhelming the primary objective.

Through repeated episodes and on‐policy updates, the RA sampler is progressively refined to produce molecules that jointly respect the target scaffold distribution, satisfy drug‐likeness expectations, and exhibit improved pharmacophoric complementarity. In this way, RA ensures stable generative dynamics, while A2C imposes adaptive, goal‐oriented pressure. Together, they form a decoupled yet cooperative framework for multi‐objective molecular generation.

### 4.5 Selection of Representative Molecules

To analyze the detailed interactions between the generated molecules and their target, representative compounds were selected based on docking score, structural novelty, and synthetic accessibility for in‐depth analysis. Firstly, generated molecules possessing a docking score below −8.0 kcal/mol, a QED greater than 0.6, and an SA score greater than 0.6 were selected to further analyze their structural diversity and binding potential [37]. The molecules with the most favorable docking conformations were clustered into 20 groups via the k‐means algorithm applied to their ECFP fingerprints. The densely populated clusters reflect dominant modes of the learned generative distribution and higher model confidence. Therefore, representative molecules were selected in proportion to cluster population: densely populated clusters contribute more representative molecules, while sparser clusters contribute fewer. The binding modes of these representatives with the target were evaluated through structure‐based molecular docking using Schrödinger Glide (2024) under the standard precision protocol, and the resulting poses were examined using the built‐in protein‐ligand interaction analysis tools [43, 45, 62]. Subsequently, MM/GBSA binding free energy calculations were further performed on the top‐ranked poses [63]. The computational method of MM/GBSA calculation is described in Section S4.

### 4.6 Evaluation Metrics

The generated molecules were evaluated using multiple metrics: (1) Validity is the percentage of chemically valid molecules among all generated molecules. Valid molecules can be sanitized by RDKit version 2023.03. (2) Uniqueness is the percentage of canonical SMILES of molecules not repeated among all generated molecules. The canonical SMILES are generated with RDKit. (3) Novelty is determined by the percentage of molecules different from those in the training dataset. (4) Diversity is the average pairwise Tanimoto‐dissimilarity between all generated molecules for a target [64]. (5) QED measures drug‐likeness and (6) SA score estimates the synthetic accessibility of generated molecules. (7) Vina score directly estimates the binding affinity of the generated molecules by Autodock Vina 1.2.0. (8) Success rate represents the percentage of molecules that pass certain criteria (QED > 0.4, SA > 0.6). The evaluation criteria are adapted from previous studies, which specify that designed molecules must exhibit drug‐like properties and synthetic accessibility [65]. (9) Scaf_sim calculates sequence similarity of the BM scaffold between generated molecules and the reference sets. We define the upper bound of the 0.3–0.4 similarity window as the ideal regime for scaffold hopping, which ensures sufficient pharmacophoric conservation while facilitating a meaningful transition in core chemical architecture. (10) Docking score estimates the binding affinity of generated molecules based on Glide Standard Precision (SP) from Schrödinger 2024 software. (11) BBB penetrant is the percentage of generated molecules with BBB permeability.

This work is financially supported by the Science and Technology Development Fund of Macao SAR (Grant Number. 0043/2023/AFJ and 0012/2025/ASJ).

## Conflicts of Interest

The authors declare no conflict of interest.

## Supporting information




**Supporting File**: advs75674‐sup‐0001‐SuppMat.docx.

## Data Availability

The data that support the findings of this study are openly available in the ChEMBL 34 database [35] (https://www.ebi.ac.uk/chembl/), the ZINC 20 database [36] (https://zinc20.docking.org/), and the BindingDB database [55] (https://www.bindingdb.org/rwd/bind/index.jsp).
