# Supplementary material for: Diffusion‐Based Generative Model With Scaffold‐Hopping Strategy Yields Highly Potent Bioactive Molecules
Source: Adv Sci (Weinh). 2026 May 15;13(44):e75674. doi: 10.1002/advs.75674 (PMC13335808; doi:10.1002/advs.75674)
Supplement: Supplementary file 1 [file ADVS-13-e75674-s001.docx]

Supporting Information

**Diffusion-Based Generative Model with Scaffold-Hopping Strategy Yields Highly Potent Bioactive Molecules**

*Yuwei Yang^1^, Xiaoqing Gong^1^, Shukai Gu^1^, Jing Li^1^, Bo Liu^1^, Yanan Tian^1^, Qianqian Zhang^1^, Xiaojun Yao^1,*^, Huanxiang Liu^1,*^.*

^1^Faculty of Applied Sciences, Macao Polytechnic University, Macao SAR, 999078, China.

^*^Corresponding: hxliu@mpu.edu.mo (Huanxiang Liu) or xjyao@mpu.edu.mo (Xiaojun Yao)

Contents

[Supplementary Methods	2](#_Toc227275056)

[SM. 1 Active Data Process and Docking Setting	2](#_Toc227275057)

[SM. 2 Pharmacophore Hypothesis Construction and Matching Coefficient Calculation	2](#_Toc227275058)

[SM. 3 BBB Predictor Architecture	4](#_Toc227275059)

[SM. 4 Computational Methods of MM/GBSA Calculations	4](#_Toc227275060)

[SM. 5 Chemical Synthesis and Characterization of Experimental Molecules lrrk2_m_1001, lrrk2_m_3364, and lrrk2_m_3570	5](#_Toc227275061)

[SM. 6 Kinase Inhibition Assay	8](#_Toc227275062)

[Supplementary Figures	10](#_Toc227275063)

[Supplementary Tables	24](#_Toc227275064)

[Supplementary References	28](#_Toc227275065)

## Supplementary Methods

### SM. 1 Active Data Process and Docking Setting

In this study, SMarT-Diff was applied to generate potential drug candidates across multiple therapeutic targets, including leucine-rich repeat kinase 2 (LRRK2), hematopoietic progenitor kinase 1 (HPK1), glucagon-like peptide-1 (GLP-1) receptor, known as GLP-1R, and the dual-target glycogen synthase kinase-3 beta (GSK3β) and c-Jun N-terminal kinase 3 (JNK3). Activity data for each target protein were collected from the BindingDB database^[1]^ and subjected to a systematic preprocessing workflow. This included the removal of duplicate entries, normalization of molecular representations (e.g., canonical SMILES conversion), and exclusion of compounds with missing or inconsistent activity annotations. Active compounds were further filtered according to IC50 values, retaining only those with IC50 < 1,000 nM, as the study focuses on inhibitor design. The resulting non-redundant active molecules (summarized in Supplementary Table 1) were compiled into an active molecule library to serve as a reference for subsequent analyses.

To investigate protein-ligand interactions, molecular docking was performed to obtain the corresponding complex structures. Preparation of the receptor pocket was therefore required. For the LRRK2 target, the co-crystal structure with PDB ID 8FO7 ^[2]^was selected. Non-essential chains were removed, followed by hydrogen addition, hydrogen-bond optimization, water removal, and restrained minimization, all performed under default parameters in the protein preparation workflow^[3]^. The docking grid was defined based on the crystallographic ligand position, with a bounding box of 15 Å. Small-molecule ligands were preprocessed using Epik to generate ionization states at neutral pH and enumerate up to 32 possible stereoisomers per compound. Docking was then conducted with Glide in SP mode, with all other parameters set to default. The same receptor and ligand preparation pipeline was applied to HPK1, GLP1R and GSK3β/JNK3 targets. For HPK1 target, the co-crystal structure with PDB ID 7M0M^[4-5]^ was selected. The co-crystal structure with PDB ID 7S15 was selected for GLP1R^[6]^. Besides, we used the protein co-crystal structures with PDB IDs 6Y9S^[7]^ and 7KSK^[8]^ as raw data for the GSK3β and JNK3 targets.

### SM. 2 Pharmacophore Hypothesis Construction and Matching Coefficient Calculation

#### (1) Pharmacophore Hypothesis Construction

To construct the reference pharmacophore hypothesis graphs, we first download bioactive molecules to the specific target from BindingDB dataset. In this study, we prefer clustered active compounds into five groups using the K-means algorithm, and the most potent molecule from each cluster was selected to construct representative pharmacophore graph. The pharmacophores hypothesis in references also need to present known key interactions with residues. Following the protocol of PGMG^[9]^, each pharmacophore hypothesis was encoded as a weighted complete graph $\mathcal{G=(V, E,}T, D)$ to construct a pharmacophore reference dataset, where nodes $v \in V$ corresponded to typed pharmacophore points and edges $e_{ab}\in E$ represented the shortest-path distances between features $a$ and $b$. The discrete node types included aromatic rings (AROM), cations (POSC), hydrogen bond acceptors (HACC), hydrogen bond donors (HDON), hydrophobic rings (HYBL), and non-ring hydrophobic centers (LHYBL). Edge weights $D$ were defined as:

$$\begin{aligned} D_{a, b}=D_{inter}\left( a, b \right)+D_{intra}\left( a \right)+D_{intra}\left( b \right)\#\left( 1 \right) \end{aligned}$$

where $D_{inter}\left( a, b \right)$ denoted the length of the shortest bond path between features (with weighting factors: single bond = 1.00, double bond = 0.87, aromatic bond = 0.91, triple bond = 0.78), and $D_{intra}\left( a \right)$ and $D_{intra}\left( b \right)$ accounted for feature radii; for ring systems, the intramolecular distance is equal to $0.2\times N_{heavy}$.

The resulting pharmacophore graphs were stored as DGL objects^[10]^, thereby forming a reference pharmacophore dataset benefit for both model conditioning and evaluation. Categorical node types were encoded as one-hot vectors and projected into fixed-dimensional embeddings, while edge weights were normalized and treated as continuous edge attributes. Consequently, five representative pharmacophore graphs were obtained for each target, ensuring diverse yet pharmacologically relevant conditioning signals.

For the dual-target lead optimization task, our pharmacophore fusion strategy is centered on maintaining essential pharmacophores across different targets, where a pharmacophore is conceptualized as a specific set of protein-ligand interactions (PLIs). We developed a specialized module that identifies and preserves core fragments embodying these critical pharmacophores in active compounds^[11]^. The module operates in two modes: an automatic extraction pipeline that captures pharmacophore-rich fragments, and a guided selection mode that incorporates expert input, allowing the prioritization of interactions involving key protein residues. In this study, the binding mode analysis in the co-crystal structures are follows the previous studies^[12-13]^, which emphasized the contribution of PLIs associated with key residues VAL135 in GSK3β^[7]^ and MET149 in JNK3^[8]^.

#### (2) Pharmacophore Matching Coefficient Calculation

We calculate a pharmacophore matching coefficient between generated molecules and a reference hypothesis by using PGMG’s matching score implementation^[9]^. Specifically, it assigns a score based on whether the spatial arrangement of these features in the molecule conforms to the shortest-path distance constraints derived from the reference pharmacophore model. For a query molecule, candidate features compatible with $\mathcal{G}_{ref}$ are extracted under the same definition, and a type-consistent assignment $\pi:= \mathcal{V}_{ref}\longrightarrow\cup_{i}\Phi_{i}$is enumerated over the family-grouped candidate pools, with null placeholders admitted for unmatched reference nodes to penalise incomplete coverage. For each alignment $\pi$, the topological distance $d_{ij}^{\pi}$ between every pair of mapped query features is compared with the corresponding reference edge $d_{ij}^{\text{ref}}$, and a pairwise match is declared successful, with $\tau=1.21$ chosen to accommodate bond-length variance from heteroatom or ring-size substitution without admitting topologically implausible alignments. The final pharmacophore matching score is then defined as

$$\begin{aligned} S^{*}=\max_{\pi\in\Pi} \frac{N_{correct}\left( \pi\right)}{N_{correct}\left( \pi\right)+ N_{error}\left( \pi\right)}\in\left[ 0,1 \right]\#\left( 2 \right) \end{aligned}$$

with early termination triggered when $S^{\star}=1$ to bound the combinatorial cost. Higher values indicate stronger 3D-pharmacophoric congruence with the target reference, and $S^{\star}$is subsequently used as the primary reward signal driving the A2C-guided sampling loop. The scoring function measures both the presence of the required chemical features and the consistency of their relative positions, ensuring that the generated structure not only contains the correct feature types but also maintains the correct geometric relationships. We adapt this encoding to our scaffold-hopping setting, where the graph serves both as a generative constraint and an evaluation target for multi-objective design. It demonstrates that encoding pharmacophore hypotheses as weighted graphs and conditioning a generator on these graphs yields valid, unique, and novel molecules aligned with target pharmacophores.

### SM. 3 BBB Predictor Architecture

A BBB permeability predictor was constructed using MoleculeNet^[14]^ training data within a Vision-LSTM regression framework^[15]^. Molecular SMILES were first converted to graph representations and then encoded as 2D grid-like images. Convolutional layers extracted local chemical patterns, while LSTM layers captured long-range interatomic dependencies and global structural motifs, enabling superior modeling of complex molecular properties compared with conventional graph neural networks.

Node and edge features were embedded and arranged on the grid for convolutional processing. Hierarchical feature maps were sequentially processed by LSTM layers, followed by a fully connected regression head with dropout to predict continuous BBB scores. The model was trained with batch size 128 for 20 epochs using the Adam optimizer (learning rate $1\times{10}^{-4}$, weight decay 0.0001), with early stopping based on validation performance. Training outcomes, including convergence and predictive accuracy, are reported in Supplementary Figure S1, demonstrating the framework’s effectiveness for BBB permeability prediction.

### SM. 4 Computational Methods of MM/GBSA Calculations

To estimate the binding free energies between molecules and proteins in each system, conventional molecular dynamics (MD) simulations were performed using the AMBER 20 software. Protein and molecule parameters were assigned using the ff14SB and gaff force fields, respectively. Each complex was solvated in the TIP3P water box, and counterions (Na+ or Cl-) were added to each system to neutralize each system. Initially, the systems were subjected to energy minimization, consisting of 5,000 steps of steepest descent followed by 2,500 steps of conjugate gradient minimization. Subsequently, the systems were gradually heated from 0 K to 300 K over 100 ps under the NVT ensemble, while the complex was restrained with a force constant of 2.0 kcal/(mol·Å²). Equilibration was then performed under the NPT ensemble in six stages. In the first five stages, harmonic restraints with force constants of 2.0, 1.5, 1.0, 0.5, and 0.1 kcal/(mol·Å²), respectively, were applied to the complex for 100 ps at each stage. In the sixth stage, all restraints were released. After equilibration, a 30 ns unrestrained MD simulation was performed. Finally, 1,000 snapshots were extracted from the final 10 ns of the trajectory, and the binding free energy was computed using the molecular mechanics generalized born surface area (MM-GBSA). Due to the high computational cost, the entropy contribution was not included in the final free energy calculation.

### SM. 5 Chemical Synthesis and Characterization of Experimental Molecules lrrk2_m_1001, lrrk2_m_3364, and lrrk2_m_3570

#### Scheme 1. The Synthetic Route for lrrk2_m_1001^a^

^a^Reagents and conditions: (a) Pd(dppf)Cl_2_, K_3_PO_4_, THF, 80 ºC, 24h under N_2_; (b) Davephos Pd G4, Cs_2_CO_3_, dioxane, 100 ℃, 4h under N_2_; (c) HCL/1,4-dioxane, rt, 16h.

K₂CO₃, dioxane, Pd(PPh_3_)_4_, 80 ºC, 16h under N_2_.

##### **General Preparation of Compound lrrk2_m_1001**

Step a: To a suspension of compound S2-1 (1.00 g, 3.48 mmol), compound S2-2 (299 mg, 3.48 mmol) and K_3_PO_4_ (1.84 g, 8.7 mmol) in THF (10 mL) was added Pd(dppf)Cl_2_ (256 mg, 0.35 mmol), then the mixture was stirred at 80 ºC for 24 hrs under N_2_. Then the reaction mixture was poured into water (50 mL), extracted with EA (50 mL*3). The combined organic phases were washed with brine (50 mL), dried over anhydrous sodium sulfate, filtered and concentrated in vacuum. The resulted residue was purified by column chromatography on silica gel (PE: EA=10:1) to give compound S2-3 (600 mg, yield: 59%) as a white solid.

LCMS (ESI): m/z 238.2 (M+H) ^+^; RT= 2.04 min.

Step b: To a suspension of compounds S2-3 (500 mg, 1.70 mmol), S2-4 (312 mg, 1.70 mmol) and Cs_2_CO_3_ (1.66 g, 5.12 mmol) in dioxane (10 mL) was added Davephos Pd G4 (133 mg, 0.17 mmol), then the mixture was stirred at 100 ºC for 4 hrs under N_2_. Then the reaction mixture was poured into water (50 mL), extracted with EA (50 mL*3). The combined organic phases were washed with brine (50 mL), dried over anhydrous sodium sulfate, filtered and concentrated in vacuum. The resulted residue was purified by column chromatography on silica gel (DCM: MeOH=10:1) to give compound S2-5 (30 mg, yield: 4%) as a white solid.

LCMS (ESI): m/z 441.1 (M+H) ^+^; RT= 1.28 min.

Step c: The mixture of compound S2-5 (30 mg, 0.068 mmol) in HCl/1,4-dioxane (4 mL) was stirred at room temperature for 16 hours. The mixture was concentrated under reduced pressure. The resulted residue was purified by prep-HPLC (0.1% NH_4_HCO_3_ in water, 20-80%MeCN) to give compound lrrk2_m_1001 (8.5 mg, yield: 53.31%) as a white solid.

LCMS (ESI): m/z 241.2 (M+H) ^+^; RT= 1.45 min.

^1^H NMR (500 MHz, DMSO) δ 12.31 (s, 1H), 11.20 (s, 1H), 8.74 (s, 1H), 7.89 (s, 1H), 7.55 (s, 1H), 7.02 (dd, J = 3.5, 2.2 Hz, 1H), 6.51 (m, J = 3.5, 1.8 Hz, 1H), 2.34 (m, J = 8.2, 4.5 Hz, 1H), 1.18 – 1.12 (m, 2H), 1.08 – 1.02 (m, 2H).

##### **NMR and HPLC Spectra of lrrk2_m_1001**

The H^1^-NMR and HPLC characterizations for compound lrrk2_m_3364 are given in Supplementary Figure S6.

#### Scheme 2. The Synthetic Route for lrrk2_m_3364^a^

^a^Reagents and conditions: (a) CuI, K_3_PO_4_, DMSO, 120 ºC, 48h under N_2_; (b) NIS, rt, 2h; (c) Suzuki, 100 ºC, 16h.

##### **General Preparation of Compound lrrk2_m_3364**

Step a: To a suspension of compounds S1-1 (2.00 g, 10.15 mmol), S1-2 (1.43 g, 15.23 mmol) and K_3_PO_4_ (6.46 g, 30.45 mmol) in DMSO (10 Ml) was added CuI (194 mg, 1.02 mmol) and PMPBO (351 mg, 1.02 mmol), then the mixture was stirred at 120 ºC for 48 hrs under N_2_. Then the reaction mixture was poured into water (50 Ml), extracted with EA (50*3). The combined organic phases were washed with brine (50 Ml), dried over anhydrous sodium sulfate, filtered and concentrated in vacuum. The resulted residue was purified by combo-flash (NH_4_HCO_3_) to give compound S1-3 (40 mg, yield: 1.88%) as a yellow solid.

LCMS (ESI): m/z 211.1 (M+H)^+^; RT= 1.74 min.

Step b: To a solution of compound S1-3 (40 mg, 0.19 mmol) and KOH (21 mg, 0.38 mmol) in DMF (2 Ml) was added I_2_ (51 mg, 0.20 mmol), the mixture was stirred at room temperature for 2 hrs. Then water (10 Ml) was added, extracted with ethyl acetate (10 Ml x 3), the combined organic phases was washed with brine (10 Ml), dried over anhydrous sodium sulfate, filtered and concentrated in vacuum to give compound S1-4 (35 mg, yield 54.8%) as a yellow solid.

LCMS (ESI): m/z 337.0 (M+H)^+^; RT= 1.91 min.

Step c: To a solution of compounds S1-4 (30 mg, 0.09 mmol), S1-5 (17 mg, 0.14 mmol) and Cs_2_CO_3_ (73 mg, 0.23 mmol) in 1,4-dioxane (2 Ml) and water (0.2 Ml) was added Pd(dppf)Cl2 (5 mg), the mixture was stirred at 100 ºC for 16 hrs. Then water (10 Ml) was added, extracted with ethyl acetate (10 Ml x 3), the combined organic phases was washed with brine (10 Ml), dried over anhydrous sodium sulfate, filtered and concentrated in vacuum. The resulted residue was purified by prep-HPLC (NH_4_HCO_3_) to give 20 mg of compound and purified again by prep-HPLC (FA) to give compound lrrk2_m_3364 (10.3 mg, yield 39.9%) as a white solid.

LCMS (ESI): m/z 507.3 (M+H)^+^; RT= 1.77 min.

^1^H NMR (400 MHz, DMSO-d_6_) δ: 13.68 (s, 1H), 8.65 (d, J = 5.8 Hz, 2H), 7.95 (d, J = 5.9 Hz, 2H), 7.86 (d, J = 1.5 Hz, 1H), 7.71 (d, J = 9.0 Hz, 1H), 7.36 (t, J = 7.9 Hz, 2H), 7.22 (dd, J = 8.9, 1.9 Hz, 1H), 7.09 (t, J = 7.3 Hz, 1H), 6.99 (d, J = 8.0 Hz, 2H).

##### **NMR and HPLC Spectra of lrrk2_m_3364**

The H^1^-NMR and HPLC characterizations for compound lrrk2_m_3364 are given in Supplementary Figure S7.

#### Scheme 3. The Synthetic Route for lrrk2_m_3570^a^

^a^Reagents and conditions: (a) Pd(PPh_3_)_4_, K₂CO₃, dioxane, 80 ºC, 16h under N_2_; (b) Pd(PPh_3_)_4_, K₂CO₃, dioxane, 100 ºC, 16h under N_2_; (c) EtOH, PPTS, 100 ºC, 16h under N_2_.

##### **General Preparation of Compound lrrk2_m_3570**

Step a: To a suspension of compound S3-1 (550 mg, 2.79 mmol), compound S3-2 (838 mg, 13.95 mmol) and K_2_CO_3_ (1.54 g, 11.2 mmol) in dioxane (10 mL) and H_2_O was added Pd(PPh_3_)_4_ (323 mg, 0.28 mmol), then the mixture was stirred at 80 ºC for 16 hrs under N_2_. Then the reaction mixture was poured into water (50 mL), extracted with EA (50 mL*3). The combined organic phases were washed with brine (50 mL), dried over anhydrous sodium sulfate, filtered and concentrated in vacuum. The resulted residue was purified by prep-HPLC (0.1% NH_4_HCO_3_ in water, 20-80%MeCN) to give compound S3-3 (85 mg, yield: 17%) as a yellow solid.

LCMS (ESI): m/z 178.2 (M+H)^+^; RT= 1.49 min.

Step b: To a suspension of compound S3-3 (550 mg, 2.79 mmol), compound S3-4 (838 mg, 13.95 mmol) and K_2_CO_3_ (1.54 g, 11.2 mmol) in dioxane (10 mL) and H_2_O (1 mL) was added Pd(PPh_3_)_4_ (323 mg, 0.28 mmol), then the mixture was stirred at 100 ºC for 16 hrs under N_2_. Then the reaction mixture was poured into water (50 mL), extracted with EA (50 mL*3). The combined organic phases were washed with brine (50 mL), dried over anhydrous sodium sulfate, filtered and concentrated in vacuum. The resulted residue was purified by prep-HPLC (0.1% NH_4_HCO_3_ in water, 10-100% MeCN) to give compound S3-5 (30 mg, yield: 17%) as a yellow solid.

LCMS (ESI): m/z 378.1 (M+H)^+^; RT= 2.41 min.

Step c: To a solution of compound S3-5 (30 mg, 0.08 mmol) in EtOH (10 mL) and H_2_O (1 mL) was added PPTS (100 mg, 0.4 mmol), then the mixture was stirred at 100 ºC for 16 hrs under N_2_. Then the reaction mixture was poured into water (50 mL), extracted with EA (50 mL*3). The combined organic phases were washed with brine (50 mL), dried over anhydrous sodium sulfate, filtered and concentrated in vacuum. The resulted residue was purified by prep-HPLC (0.1% NH_4_HCO_3_ in water, 10-70%MeCN) to give compound lrrk2_m_3570 (5.2 mg, yield: 23%) as a yellow solid.

LCMS (ESI): m/z 294.2 (M+H)^+^; RT=1.63 min.

^1^H NMR (400 MHz, DMSO) δ 12.91 (s, 1H), 10.39 (s, 1H), 8.71 (s, 1H), 8.27 (s, 1H), 7.96 (s, 1H), 7.66 (d, J = 8.7 Hz, 1H), 7.35 (dd, J = 8.7, 1.7 Hz, 1H), 2.54 (d, J = 0.8 Hz, 3H).

^19^F NMR (376 MHz, DMSO) δ -58.97 (s).

##### **NMR and HPLC spectra of lrrk2_m_3570**

The H^1^-NMR and HPLC characterizations for compound lrrk2_m_3364 are given in Supplementary Figure S8.

### SM. 6 Kinase Inhibition Assay

Kinase inhibition assays were performed using the ADP-Glo^TM^ Kinase Assay (Promega) in 384-well format. A 2× ATP/substrate solution and a 2× kinase solution was prepared in kinase reaction buffer. Compounds were dispensed into the assay plates (100 nL per well) using an Echo 655 acoustic dispenser. Subsequently, 5 μL of the 2× kinase solutions were added to each well, followed by centrifugation at 1000 rpm for 1 min and incubation at 25 °C for 10 min. The kinase reactions were initiated by the addition of 5 μL of the 2× ATP/substrate solution. Plates were centrifuged at 1000 rpm for 1 min and incubated at 25 °C for 120 min (LRRK2 G2019S) or 60 min (wild-type LRRK2).

Following the kinase reaction, 5 μL of ADP-Glo reagent was added to each well. Plates were centrifuged at 1000 rpm for 1 min and incubated at 25 °C for 60 min to terminate the reaction and deplete unconsumed ATP. Subsequently, 10 μL of the Kinase Detection Reagent was added, followed by centrifugation (1000 rpm, 1 min) and incubation at 25 °C for 60 min. Luminescence signals were recorded using a BMG plate reader.

#### Data Analysis

The percentage inhibition (inhibition%) was calculated as:

$$\begin{aligned} Inhibition\%=100\times\frac{ave High control - compound well}{ave High Control - ave Low Control}\#\left( 3 \right) \end{aligned}$$

Assay performance was assessed using standard statistical parameters, including signal-to-background ratio (S/B), coefficient of variation (CV%) of high and low controls, and the Z’ factor:

$$\begin{aligned} S/B=\frac{ave High Control}{ave Low Control}\#\left( 4 \right) \end{aligned}$$

$\begin{aligned} CV\%\left( Low Control \right)=100\times\left( \frac{SD Low control}{ave Low control} \right)\#\left( 5 \right) \end{aligned}$

$$\begin{aligned} CV\%\left( High Control \right)=100\times\left( \frac{SD High control}{ave High control} \right)\#\left( 6 \right) \end{aligned}$$

$$\begin{aligned} Z’=1-3\times\frac{\left( SDLowcontrol+SDHighcontrol \right)}{\left( aveHighcontrol-aveLowcontrol \right)}\#\left( 7 \right) \end{aligned}$$

IC_50_ values were determined by nonlinear regression of the inhibition% versus the logarithm of compound concentrations using a four-parameter sigmoidal dose–response model (variable slope) in GraphPad Prism 10.0. The fitting equation was:

$\begin{aligned} Y=Bottom+\frac{Top-Bottom}{1+{10}^{\left( LogIC50-X \right)\times HillSlope}}\#\left( 8 \right) \end{aligned}$

where *X* is the logarithm of the compound concentration, *Y* is the percentage inhibition, *Top* and *Bottom* represent the upper and lower plateaus (in the same units as *Y*), *logIC₅₀* is the midpoint, and *HillSlope* is the slope factor of the curve.

## Supplementary Figures

**Figure S1**. **Training performance of the BBB predictor.**

From left to right: epoch-wise decrease in training loss and the corresponding evolution of training and test accuracy.

**Figure S2**. **Multi-objective trade-offs across ablation variants of SMarT-Diff.**

**(A)** Comparison of six model configurations across five metrics: scaffold similarity to the reference set (Scaf_sim; target near 0.4), internal chemical diversity (IntDiv), and the mean QED, docking and SA scores. (Each variant in colored polygon, RA-only baseline in grey dashed polygon) **(B)** Distributions of QED, SA and docking scores for active reference molecules, RA sampled molecules and A2C sampled molecules.

**Figure S3. Scaffold-level out-of-distribution performance of SMarT-Diff.**

(A) Summary of scaffold novelty for molecules generated by SMarT-Diff (n = 10,000). (B) Fingerprint-space distribution of Bemis & Murcko (BM)^[18]^ scaffolds from the training set (green), reference actives (blue) and molecules generated by SMarT-Diff (red). (C) Distributions of the nearest-neighbor BM-scaffold Tanimoto similarity of SMarT-Diff generated molecules to the training set (green) and reference actives (blue). (D) Scaffold novelty versus binding affinity for the drug-likeness-filtered subset of SMarT-Diff-generated molecules.

**Figure S4**. **The t-SNE visualization of molecules generated by SMarT-Diff and active inhibitors for the single-target design tasks of (A) HPK1 and (B) LRRK2.**

**Figure S5**. **Top ten selected LRRK2 inhibitors generated by SMarT-Diff.**

**Figure S6**. **Structural verification of compound lrrk2_m_1001.**

(A) ^1^H-NMR spectrum of lrrk2_m_1001.

(B) LC-MS spectrum of lrrk2_m_1001 confirming the expected molecular ion peak.

**Figure S7. Structural verification of compound lrrk2_m_3364.**

(A) ^1^H-NMR spectrum of lrrk2_m_3364.

(B) LC-MS spectrum of lrrk2_m_3364 confirming the expected molecular ion peak.

**Figure S8**. **Structural verification of compound lrrk2_m_3570.**

(A) ^1^H-NMR spectrum of lrrk2_m_3570.

(B) LC-MS spectrum of lrrk2_m_3570 confirming the expected molecular ion peak.

**Figure S9. IC_50_ curves of the compounds measured by ADP-Glo^TM^ Kinase Assay.**

(**A**) Positive control LRRK2-IN-1 (IC_50_: 3.141 nM for LRRK2 and IC_50_: 5.250 nM for LRRK2 G2019S), (**B**) Experimental Molecule lrrk2_m_1001 (IC_50_: 1.544 nM for LRRK2 and IC_50_: 2.335 nM for LRRK2 G2019S), (**C**) Experimental Molecule lrrk2_m_3364 (IC_50_: 66.83 nM for LRRK2 and IC_50_: 54.24 nM for LRRK2 G2019S), (**D**) Experimental Molecule lrrk2_m_3570 (IC_50_: 16.404µM for LRRK2 and IC_50_: 31.537 µM for LRRK2 G2019S).

Ten different concentrations were tested for each compound. Compounds lrrk2_m_3364 and lrrk2_m_3570 were prepared using a 3-fold dilution serial dilution starting from 30 µM, compound lrrk2_m_1001 was prepared using a 3-fold dilution starting from 100 nM, and the positive control was assayed in a dilution series starting from 1 µM. Residual kinase activity was determined based on luminescence readouts, and the data were normalized to vehicle (100% activity) and complete inhibition controls (0% activity). IC_50_ values were calculated by nonlinear regression using a sigmoidal dose-response model (variable slope), with the top and bottom parameters constrained to 100% and 0%, respectively. Curves were fitted with least-squares regression using 10 data points per compound (n = 10). The dashed vertical lines indicate the calculated IC_50_ values. The x-axis shows the molar concentration of the compounds, and the y-axis represents the percentage of residual kinase activity.

**Figure S10. Performance of generated molecules targeting GLP-1R.** (A) Density distributions of QED, SA score and docking score for generated molecules (copper) versus reference GLP-1R actives (moss); (B) 2D structures of the six represented generated molecules with their QED, SA and docking score.

**Figure S11**. **The t-SNE visualization of SMarT-Diff’s generated molecules and active inhibitors for the dual-target inhibitor design task on GSK3β and JNK3.**

**Figure S12**. **The MM/GBSA binding free energies of 20 SMarT-Diff generated molecules (brick blue) with active ligands in the co-crystal structure of GSK3β and JNK3.**

## Supplementary Tables

**Table S1**. **Number of active compounds for different targets.**

| **Target** | **Number of Active Compounds** |
| --- | --- |
| LRRK2 | 8512 |
| HPK1 | 3166 |
| GSK3β | 2128 |
| JNK3 | 791 |

**Table S2**. **Ablation results about SMarT-Diff with RA sampling**.

| **Model**  **Description** | **Validity↑** | **Unique@10000^a^↑** | **FCD^b^↑** | **Success rate^c^↑** | **IntDiv.^d^↑** | **Novelty↑** | **Nspdk^e^↓** | **Scaf_sim^f^** |
| --- | --- | --- | --- | --- | --- | --- | --- | --- |
| Baseline (SGM) with no condition | 0.852 | 0.999 | 17.914 | 0.060 | 0.900 | 1.000 | 0.034 | 0.032 |
| + DiT backbone | 0.419 | **1.000** | 31.792 | 0.008 | **0.905** | 1.000 | 0.076 | **0.339** |
| + Scaffold graph conditioning | 0.953 | 0.786 | 19.665 | 0.312 | 0.861 | 1.000 | 0.052 | 0.430 |
| + RA sampling | 0.943 | 0.861 | 19.525 | 0.316 | 0.861 | 1.000 | 0.051 | 0.732 |
| + Enhance edge embedding | 0.906 | 0.793 | 18.810 | 0.357 | 0.861 | 1.000 | 0.050 | 0.961 |
| + QED / SA conditioning (DiT) | 0.934 | 0.797 | 18.651 | 0.392 | 0.863 | 1.000 | **0.048** | 0.794 |
| SMarT-Diff_RA | 0.936 | 0.791 | 19.260 | **0.394** | 0.863 | 1.000 | 0.050 | 0.654 |

^a^ Uniqueness in 10000 generated molecules.

^b^ Fréchet ChemNet Distance (FCD) measures diversity of the set between the distribution of generated molecules and training molecules^[16]^.

^c^ Success rate in ablation studies means the proportion of generated molecules with QED > 0.6 and SA score > 0.6.

^d^ Internal diversity (IntDiv.) measures the average pairwise distance of whole molecular structure in the generated set.

^e^ Neighborhood Subgraph Pairwise Distance Kernel (NSPDK) computes the Maximum Mean Discrepancy (MMD) between generated and test graphs based on node and edge features^[17]^.

^f^ Scaf_sim represents BM scaffold sequence similarity between generated molecules and references within the 0.3-0.4 interval (optimally near 0.4).

**Table S3**. **Ablation results about SMarT-Diff with A2C sampling based on RA-only sampling.**

| **Model**  **Description** | **Validity↑** | **Unique@**  **10000↑** | **FCD↑** | **Success rate↑** | **IntDiv.↑** | **Novelty↑** | **Nspdk↓** | **Scaf_sim** |
| --- | --- | --- | --- | --- | --- | --- | --- | --- |
| RA sampling only | **0.936** | 0.791 | 19.260 | **0.394** | 0.863 | 1.000 | 0.050 | 0.654 |
| + Actor-critic w/o conditions ^a^ | 0.911 | 0.781 | 19.066 | 0.330 | 0.876 | 1.000 | 0.044 | 0.408 |
| Action as gate coefficient | 0.658 | 0.647 | **23.969** | 0.024 | **0.927** | 1.000 | 0.128 | 0.057 |
| Cumulative discounted rewards with PMS ^b^ | 0.903 | **0.874** | 19.138 | 0.275 | 0.875 | 1.000 | 0.046 | 0.313 |
| + QED / SA conditioning (DiT) | 0.931 | 0.860 | 19.171 | 0.313 | 0.873 | 1.000 | 0.048 | 0.304 |
| + BBB (final model) | 0.932 | 0.851 | 18.702 | 0.318 | 0.870 | 1.000 | 0.046 | **0.363** |

^a^ w/o means without specific parameters.

^b^ PMS means Pharmacophore Matching Score that measure the pharmacophore alignment between generated molecules and reference.

**Table S4**. **IC50 values of the SMarT-Diff generated compounds experimented with LRRK2**.

| **Assay name** | **Compound ID^a^** | **IC_50_ (nM)** | **Hill slope^b^** |
| --- | --- | --- | --- |
| LRRK2_ADP-Glo Assay | LRRK2-IN-1 | 3.14 | 0.95 |
|  | lrrk2_m_1001 | 1.54 | 0.99 |
|  | lrrk2_m_3364 | 66.83 | 0.92 |
|  | lrrk2_m_3570 | 16404 | 0.39 |
| LRRK2 G2019S_ADP-Glo Assay | LRRK2-IN-1 | 5.25 | 1.03 |
|  | lrrk2_m_1001 | 2.34 | 1.06 |
|  | lrrk2_m_3364 | 54.24 | 1.06 |
|  | lrrk2_m_3570 | 31537 | 0.38 |

^a^ Compound ID refers to the experimental molecule ID given to the synthesized molecules.

^b^ Hill slopes higher than -0.4 indicate that the curve is not sigmoidal, or very flat, or not descending.

## Supplementary References

[1]	T. Liu, L. Hwang, S. K. Burley, C. I. Nitsche, C. Southan, W. P. Walters, M. K. Gilson, *Nucleic Acids Res* **2025**, *53* (D1), D1633, https://doi.org/10.1093/nar/gkae1075.

[2]	H. Zhu, P. Hixson, W. Ma, J. Sun, *Cell Discov.* **2024**, *10* (1), 10, https://doi.org/10.1038/s41421-023-00639-8.

[3]	G. M. Sastry, M. Adzhigirey, T. Day, R. Annabhimoju, W. Sherman, *J. Comput. Aided Mol. Des.* **2013**, *27* (3), 221, https://doi.org/10.1007/s10822-013-9644-8.

[4]	H. Ge, L. Peng, Z. Sun, H. Liu, Y. Shen, X. Yao, *Front. Pharmacol.* **2022**, *13*, 850855, https://doi.org/10.3389/fphar.2022.850855.

[5]	B. A. Vara, S. M. Levi, A. Achab, D. A. Candito, X. Fradera, C. A. Lesburg, S. Kawamura, B. M. Lacey, J. Lim, J. L. Methot, Z. Xu, H. Xu, D. M. Smith, J. A. Piesvaux, J. R. Miller, M. Bittinger, S. H. Ranganath, D. J. Bennett, E. F. DiMauro, A. Pasternak, *ACS Med. Chem. Lett.* **2021**, *12* (4), 653, <https://doi.org/10.1021/acsmedchemlett.1c00096>.

[6]	D. A. Griffith, D. J. Edmonds, J.-P. Fortin, A. S. Kalgutkar, J. B. Kuzmiski, P. M. Loria, A. R. Saxena, S. W. Bagley, C. Buckeridge, J. M. Curto, D. R. Derksen, J. M. Dias, M. C. Griffor, S. Han, V. M. Jackson, M. S. Landis, D. Lettiere, C. Limberakis, Y. Liu, A. M. Mathiowetz, J. C. Patel, D. W. Piotrowski, D. A. Price, R. B. Ruggeri, D. A. Tess, *J. Med. Chem.* **2022**, 65 (12), 8208, https://doi.org/10.1021/acs.jmedchem.1c01856.

[7]	R. Buonfiglio, F. Prati, M. Bischetti, C. Cavarischia, G. Furlotti, R. Ombrato, *Molecules* **2020**, *25* (9), 2163, https://doi.org/10.3390/molecules25092163.

[8]	Y. Feng, H. Park, L. Bauer, J. C. Ryu, S. O. K. Yoon, *ACS Med. Chem. Lett.* **2021**, *12* (1), 24, https://doi.org/10.1021/acsmedchemlett.0c00533.

[9]	H. Zhu, R. Zhou, D. Cao, J. Tang, M. Li, *Nat. Commun.* **2023**, *14* (1), 6234, https://doi.org/10.1038/s41467-023-41454-9.

[10]	D. Zheng, M. Wang, Q. Gan, X. Song, Z. Zhang, G. Karypis, presented at *Proceedings of the 14th ACM International Conference on Web Search and Data Mining*, Virtual Event, Israel, **2021**.

[11]	S. Chen, J. Xie, R. Ye, D. Xu, Y. Yang, *Chem. Sci* **2024**, *15* (27), 10366, https://doi.org/10.1039/D4SC00094C.

[12]	Y. Xie, C. Shi, H. Zhou, Y. Yang, W. Zhang, Y. Yu, L. Li, in *International Conference on Learning Representations (ICLR)* **2021**.

[13]	W. Jin, R. Barzilay, T. Jaakkola, in *International Conference on Machine Learning* **2020**.

[14]	Z. Wu, B. Ramsundar, E. N. Feinberg, J. Gomes, C. Geniesse, A. S. Pappu, K. Leswing, V. Pande, *Chem. Sci.* **2018**, *9* (2), 513, https://doi.org/10.1039/c7sc02664a.

[15]	B. Alkin, M. Beck, K. Pöppel, S. Hochreiter, J. Brandstetter, in *The Thirteenth International Conference on Learning Representations* **2025**.

[16]	K. Preuer, P. Renz, T. Unterthiner, S. Hochreiter, G. Klambauer, *J. Chem. Inf. Model.* **2018**, *58* (9), 1736, https://doi.org/10.1021/acs.jcim.8b00234.

[17]	H. Chen, C. Xu, L. Zheng, Q. Zhang, X. Lin, *IEEE Transactions on Knowledge & Data Engineering* **2024**, *36* (12), 7954, <https://doi.org/10.1109/tkde.2024.3466301>.

[18]	G. W. Bemis, M. A. Murcko, J. Med. Chem. 1996, 39 (15), 2887, https://doi.org/10.1021/jm9602928
